# Susceptibility of Mitophagy‐Deficient Tumors to Ferroptosis Induction by Relieving the Suppression of Lipid Peroxidation

**DOI:** 10.1002/advs.202412593

**Published:** 2024-12-16

**Authors:** Shan Liu, Jing‐Hong Chen, Li‐Chao Li, Zhi‐Peng Ye, Jian‐Nan Liu, Yu‐Hong Chen, Bing‐Xin Hu, Jia‐Hong Tang, Gong‐Kan Feng, Zhi‐Ming Li, Chu‐Xia Deng, Rong Deng, Xiao‐Feng Zhu, Hai‐Liang Zhang

**Affiliations:** ^1^ State Key Laboratory of Oncology in South China Guangdong Provincial Clinical Research Center for Cancer Guangdong Key Laboratory of Nasopharyngeal Carcinoma Diagnosis and Therapy Sun Yat‐sen University Cancer Center Guangzhou 510060 China; ^2^ Department of Medical Oncology The Seventh Affiliated Hospital Sun Yat‐sen University Shenzhen 518107 China; ^3^ Department of Medical Oncology Sun Yat‐sen University Cancer Center Guangzhou 510060 China; ^4^ Guangzhou Municipal and Guangdong Provincial Key Laboratory of Protein Modification and Degradation School of Basic Medical Sciences Guangzhou Medical University Guangzhou 511436 China; ^5^ Faculty of Health Sciences University of Macau Macau SAR 999078 China; ^6^ Department of Oncology The Affiliated Yantai Yuhuangding Hospital of Qingdao University Yantai 264000 China

**Keywords:** ferroptosis, mitophagy‐deficient tumors, lipid peroxidation, parkin

## Abstract

The identification of ferroptosis‐sensitive cancers is critical for the application of ferroptosis‐inducing therapies in cancer therapy. Here, patient‐derived organoid screening models of colorectal cancer are established to identify tumors that are sensitive to ferroptosis‐inducing therapy. This study discovers that patient‐derived tumors characterized by mitophagy deficiency are hypersensitive to ferroptosis‐inducing therapies. Mechanistically, a novel negative feedback regulatory pathway of lipid peroxidation is identified, which is one of the important intrinsic anti‐ferroptosis mechanisms of cancer cells. Lipid peroxidation‐mediated endoplasmic reticulum stress transcriptionally upregulates Parkin to promote mitophagy through ATF4. Mitophagy limits the generation of lipid peroxidation products and subsequently inhibits ferroptosis by inhibiting the accumulation of mitochondrial ROS. Mitophagy‐deficient tumors lack this anti‐ferroptotic mechanism, unleashing the generation of lipid peroxidation and potent ferroptotic cell death induced by erastin, RSL3, cysteine deprivation, radiotherapy, and immunotherapy. More importantly, ferroptosis‐inducing therapy selectively inhibits the growth and distant metastasis of mitophagy‐deficient tumors in vivo. In summary, patient‐derived organoids of colorectal cancer patients for screening ferroptosis‐sensitive tumors are established, providing a paradigm for identifying that patient‐derived tumors are sensitive to ferroptosis‐inducing therapies. This study concludes that mitophagy‐deficient tumors are vulnerable to ferroptosis induction, which may lead to the development of new therapeutic strategies for tumors deficient in mitophagy.

## Graphical abstract

Mitophagy defects lead to increased mitochondrial ROS in cancer cells. On the one hand, the increased mitochondrial ROS promotes tumor metastasis, and on the other hand, mitochondrial ROS accelerates the production of tumor lipid peroxidation under ferroptosis induction therapy. This makes tumors with mitophagy defects acquire the ability to progress malignantly while also showing ferroptosis vulnerability.

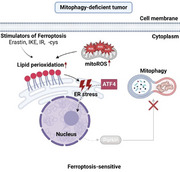

## 1 Introduction

Ferroptosis is an iron‐dependent form of programmed cell death driven by lipid peroxidation.^[^ ^1^, ^2^ ^]^ Ferroptosis inhibits the occurrence and development of tumors and promotes the curative effect of cancer therapy.^[^ ^1^, ^3^, ^4^ ^]^ The induction of ferroptosis is a promising antitumor strategy. However, the high heterogeneity of the sensitivity of tumors to ferroptosis greatly limits current translational research on ferroptosis‐inducing therapies. The identification of ferroptosis‐sensitive tumors is critical for the application of ferroptosis‐inducing therapies in cancer. Patient‐derived organoids (PDOs) are 3D cell clusters obtained from patient tumor tissues through 3D culture in vitro and play an important role in new drug screening and drug sensitivity studies.^[^ ^5^, ^6^, ^7^, ^8^ ^]^

Mitochondrial homeostasis consists of the synthesis and degradation of mitochondria and plays an important role in the malignant progression of tumors.^[^ ^9^ ^]^ Our previous study revealed that defects in mitochondrial degradation, that is, defects in mitophagy, promote the malignant progression of breast cancer.^[^ ^10^ ^]^ We found that mitophagy deficiency mediated by the low expression or deletion of ULK1 promoted NLRP3 inflammasome activation and IL‐1β secretion, leading to the formation of osteolytic lesions and the occurrence of breast cancer bone metastasis.^[^ ^10^ ^]^ Similar to our findings, multiple studies have reported that mitophagy defects are present in various tumors and contribute to tumor progression.^[^ ^11^, ^12^, ^13^, ^14^, ^15^, ^16^, ^17^ ^]^ Arajita H Chourasia et al. reported that BNIP3 deficiency‐mediated mitophagy defects enhanced Hif‐1α stabilization by increasing mitochondrial ROS, thereby promoting lung metastasis of breast cancer cells.^[^ ^16^ ^]^ BRCA1 deficiency‐mediated mitophagy defects promoted breast cancer cells to secrete IL‐1β by activating the NLRP3 inflammasome, which then promoted macrophage differentiation to the M2 phenotype, leading to CD8^+^ T cell inactivation, and ultimately enhancing breast cancer lung metastasis.^[^ ^17^ ^]^ These studies suggest that defective mitophagy is an important factor driving the malignant progression of certain tumors. However, methods of inhibiting the growth and metastasis of mitophagy‐deficient tumors remain to be studied. Mitochondria are closely related to ferroptosis. Mitochondrial morphological changes, such as mitochondrial fragmentation and crista enlargement, are among the hallmarks of ferroptosis.^[^ ^1^, ^18^, ^19^ ^]^ Mitochondrial membrane hyperpolarization driven by OXPHOS has recently been implicated in promoting ferroptosis induced by cystine deprivation or erastin treatment.^[^ ^20^ ^]^ However, the role of mitophagy in ferroptosis remains unclear. Parkin is a RING‐type E3 ubiquitin protein ligase and a key factor driving mitophagy. When mitochondria are damaged, Parkin and PINK1 are recruited to damaged mitochondria. PINK1 phosphorylates Parkin and then enhances Parkin's E3 ubiquitin ligase activity. Activated Parkin promotes the ubiquitination of mitochondrial outer membrane proteins, and then autophagy adaptors such as p62/SQSTM1, NBR1, NDP52, and OPTN bind to ubiquitin on the mitochondrial surface, thereby promoting the occurrence of mitophagy.^[^ ^21^ ^]^ Low expression of Parkin is positively correlated with the malignant progression of various tumors. ATF‐4 is a member of the activating transcription factor/cAMP response element binding protein family and a key factor in the ER stress response pathway mediated by PERK and eIF2α. ER stress inhibits the translation of most mRNAs, but selectively stimulates the translation of ATF‐4. The expression of ATF‐4 target genes is crucial for alleviating ER stress.^[^ ^22^ ^]^

In this study, we established PDO screening models of colorectal cancer to identify tumors that are sensitive to ferroptosis‐inducing therapies. We found that tumors characterized by mitophagy deficiency are sensitive to ferroptosis‐inducing therapies. Mechanistically, lipid peroxidation‐ATF4‐Parkin pathway‐mediated mitophagy limits lipid peroxidation to halt ferroptosis in cancer, and mitophagy deficiency strongly increases lipid peroxidation and enhances ferroptosis induced by erastin, RSL3, cysteine deprivation, radiotherapy or immunotherapy. Importantly, we found that ferroptosis‐inducing therapies selectively inhibited mitophagy‐deficient tumor growth and metastasis, providing new therapeutic strategies for this subtype of malignantly progressing tumors.

## 2 Results

### 2.1 Defective Mitophagy Confers PDOs Ferroptosis Sensitivity

**Figure 1:**
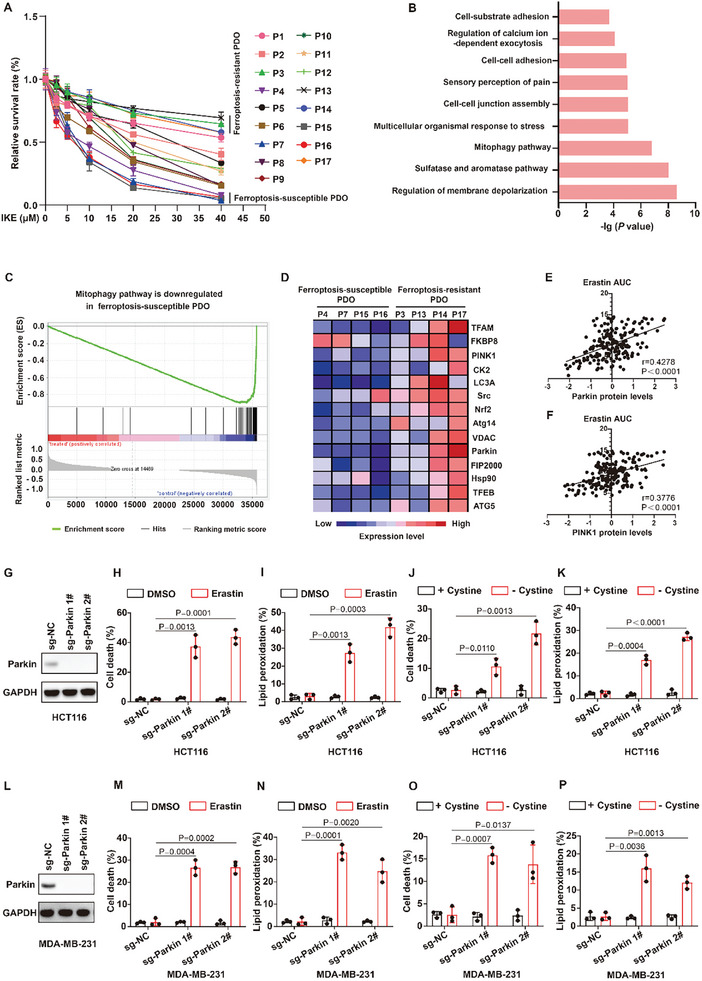
Mitophagy‐deficient colorectal cancer patients derived organoids (CRC PDOs) are sensitive to ferroptosis inducers. A) Relative survival rate in the indicated PDOs treated with imidazole ketone erastin (IKE). B) The enrichment analysis of pathways downregulated in the ferroptosis‐susceptible group compared to the ferroptosis‐resistant group. The Benjamini–Hochberg (BH) procedure was used with one‐sided P values adjusted for multiple testing. C) GSEA (Reactome) of mitophagy pathway. The BH procedure was used with two‐sided P values adjusted for multiple testing. D) Heat map of mitophagy pathway genes in ferroptosis‐susceptible PDOs and the ferroptosis‐resistant PDOs. E,F) Relationship between Parkin (E) or PINK1 (F) expression and sensitivity to ferroptosis inducer erastin in 207 cancer cell lines of different cancer types. G,L) Immunoblot showing the expression of Parkin in the indicated HCT116 cells (G) or MDA‐MB‐231 cells (L). H–K) Cell death and lipid peroxidation measurement in the indicated HCT116 cells treated with 12 µm erastin for 26 h (H,I) or cystine deprivation for 19 h (J,K). M–P) Cell death and lipid peroxidation measurement in the indicated MDA‐MB‐231 cells treated with 2 µm erastin for 12 h (M,N) or cystine deprivation for 7 h (O,P). (A) Data are the mean ± s.d.; n =  3 biologically independent experiments., Statistical analysis was performed using a two‐way ANOVA with Tukey's multiple comparisons test. (G,L), Data are representative of n =  3 biologically independent experiments. (H–K,M–P, Data are the mean ± s.d.; n =  3 biologically independent experiments. Statistical analysis was performed using an unpaired two‐tailed Student's t‐test.

To identify tumors that are sensitive to ferroptosis‐inducing therapies, we established a colorectal cancer (CRC) organoid biobank, including 17 CRC PDOs (P1 to P17 PDOs) from patients. We treated PDOs with imidazole ketone erastin (IKE), which is commonly employed as a ferroptosis inducer in vivo, and then assessed drug sensitivity. As shown in **Figure** **1**A, P4, P7, P15, and P16 PDOs were sensitive to IKE, whereas the P3, P13, P14, and P17 PDOs showed obvious resistance to IKE. To explore the characteristics of ferroptosis‐susceptible tumors, we performed transcriptomic analysis of all PDOs via RNA sequencing and compared messenger RNA profiles between ferroptosis‐susceptible groups (P4, P7, P15, and P16) and ferroptosis‐resistant groups (the P3, P13, P14, and P17). Gene Ontology (GO) analysis revealed that the mitophagy pathway was dramatically downregulated in the ferroptosis‐susceptible group (Figure 1B). In addition, we used gene sets from Reactome for gene set enrichment analysis (GSEA) to conduct gene enrichment and obtain similar results, which revealed that the mitophagy pathway was highly downregulated in the ferroptosis‐susceptible group (Figure 1C). Notably, the expression of Parkin and PINK1, important molecules of the mitophagy pathway, markedly decreased in all ferroptosis‐susceptible PDOs (Figure 1D). Furthermore, we analyzed the relationship between the expression of the mitophagy‐related proteins Parkin and PINK1 and ferroptosis sensitivity in 207 cancer cell lines from different cancer types. The results revealed that the expression of Parkin or PINK1 was negatively correlated with the sensitivity of cancer cells to the ferroptosis inducer erastin (Figure 1E,F), suggesting that cancer cells with defective mitophagy are sensitive to ferroptosis. Taken together, these results indicate that defective mitophagy may confer ferroptosis sensitivity in PDOs.

**Figure 2:**
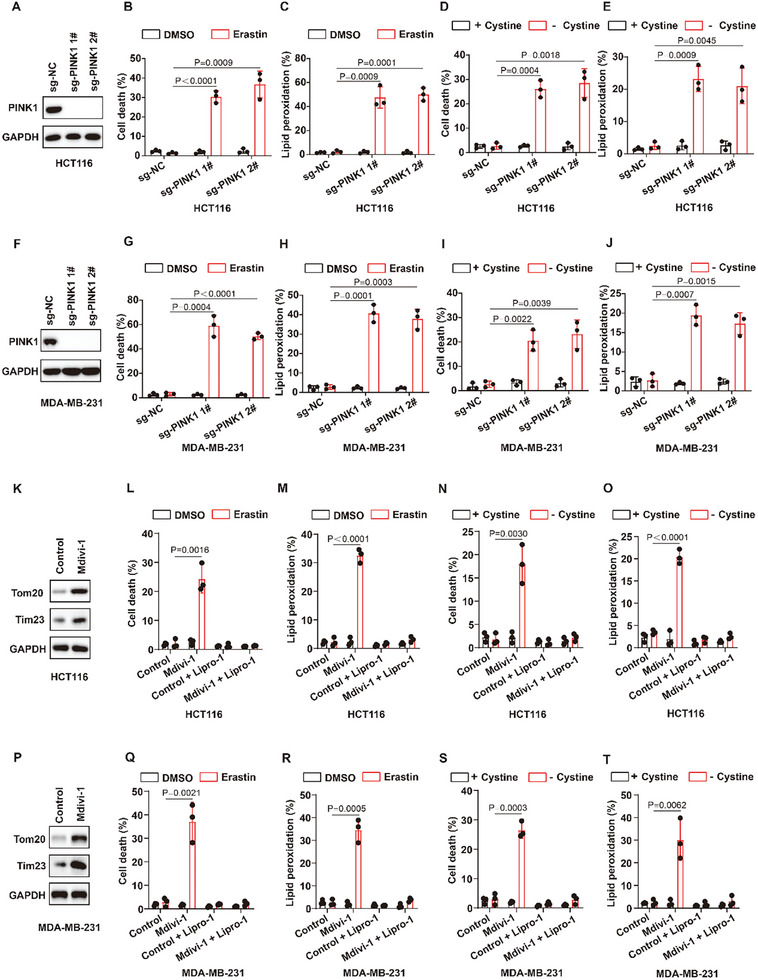
Mitophagy‐deficient cancer cells are sensitive to ferroptosis inducers. A,F) Immunoblot showing the expression of PINK1 in the indicated HCT116 cells (A) or MDA‐MB‐231 cells (F). B–E) Cell death and lipid peroxidation measurement in the indicated HCT116 cells treated with 12 µm erastin for 26 h (B,C) or cystine deprivation for 19 h (D,E). G–J) Cell death and lipid peroxidation measurement in the indicated MDA‐MB‐231 cells treated with 2 µm erastin for 12 h (G, H) or cystine deprivation for 7 h (I,J). K) Immunoblot showing the expression of Tom20 or Tim23 in HCT116 cells treated with control or mitophagy inbibitor 10 µm Mdivi‐1 for 18 h. (L‐O) Cell death and lipid peroxidation measurement in the indicated HCT116 cells treated with 12 µm erastin for 26 h (L,M) or cystine deprivation for 19 h (N,O) with or or without 10 µm Mdivi‐1 or 10 µm Lipro‐1. P) Immunoblot showing the expression of Tom20 or Tim23 in MDA‐MB‐231 cells treated with control or 10 µm Mdivi‐1 for 12 h. Q–T) Cell death and lipid peroxidation measurement in the indicated MDA‐MB‐231 cells treated with 2 µm erastin for 12 h (Q, R) or cystine deprivation for 7 h (S,T) with or or without 10 µm Mdivi‐1 or 10 µm Lipro‐1. (A,F,K,P) Data are representative of n =  3 biologically independent experiments. (B–E,G–J,L–O,Q–T, Data are the mean ± s.d.; n =  3 biologically independent experiments. Statistical analysis was performed using an unpaired two‐tailed Student's t‐test.

To validate the results of organoid screening, the mitophagy gene Parkin was knocked out in the colorectal cancer cell line HCT116 and the breast cancer cell line MDA‐MB‐231 to construct cell models of mitophagy deficiency (Figure 1G,L). The results revealed that mitophagy‐deficient colorectal cancer cells and breast cancer cells were more sensitive to ferroptosis induced by erastin or cystine deprivation or RSL3 than were wild‐type cells, as shown by significantly increased lipid peroxidation and ferroptosis (Figure 1G–P; Figure S1A–D, Supporting Information). We found that knockout of another mitophagy gene PINK1 could also significantly enhance the sensitivity of cancer cells to the ferroptosis inducers erastin or cystine deprivation or RSL3 (**Figure** **2**A–J; Figure S1A–D, Supporting Information). Consistently, inhibiting mitophagy with the mitophagy inhibitor Mdivi‐1 strongly enhanced erastin‐ or cystine deprivation‐ or RSL3‐induced ferroptosis in colorectal cancer cells and breast cancer cells (Figure 2K–T; and Figure S1E–H, Supporting Information). The ferroptosis inhibitor Liproxstatin‐1 (Lipro‐1) significantly reversed the enhancement of erastin‐ or RSL3‐ or cystine deprivation‐induced cell death mediated by mitophagy deficiency in cancer cells (Figure S1A–P, Supporting Information), indicating that mitophagy deficiency indeed promoted ferroptosis in cancer cells.

Taken together, these results demonstrate that cancers with defective mitophagy are susceptible to ferroptosis. These results also implicate mitophagy as an important regulator of ferroptosis sensitivity in human cancer cells and human tumors.

### 2.2 ATF4‐Mediated Parkin Expression Promotes Mitophagy During the Ferroptotic Process in Cancer Cells

**Figure 3:**
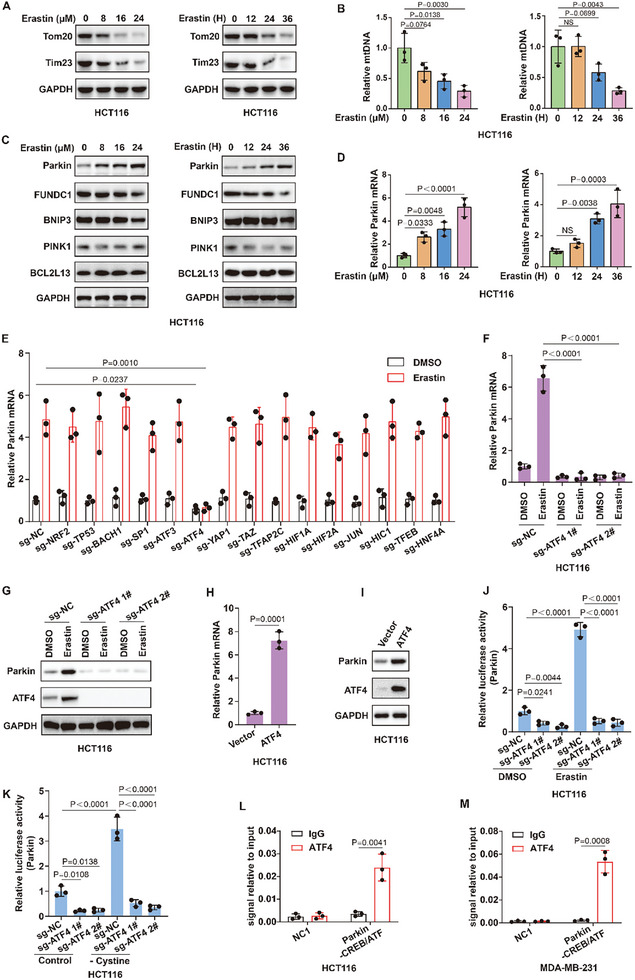
Mitophagy is enhanced during the ferroptotic process in cancer cells. A) Immunoblot showing the expression of Tom20 or Tim23 in HCT116 cells treated with erastin at the indicated concentrations and times. Left, time, 28h. Right, concentration, 16 µm. B) The relative mitochondrial DNA (mtDNA) measurement in HCT116 cells treated with erastin at the indicated concentrations and times. Left, time, 28h. Right, concentration, 16 µm. C) Immunoblot showing the expression of mitophagy‐related proteins in HCT116 cells treated with erastin at the indicated concentrations and times. Left, time, 28h. Right, concentration, 16 µm. D) QPCR showing the expression of Parkin mRNA in HCT116 cells treated with erastin at the indicated concentrations and times. Left, time, 28h. Right, concentration, 16 µm. E) Parkin mRNA measurement in the indicated HCT116 cells treated with 16 µm erastin for 22h. F) Parkin mRNA measurement in control HCT116 cells (sg‐NC) and ATF4 knockout HCT116 cells (sg‐ATF4 1# or sg‐ATF4 2#) treated with DMSO or 16 µm erastin for 22h. G) Immunoblot showing the expression of Parkin in control HCT116 cells (sg‐NC) and ATF4 knockout HCT116 cells (sg‐ATF4 1# or sg‐ATF4 2#) treated with DMSO or 16 µm erastin for 22h. H) Parkin mRNA was measured in control HCT116 cells (Vector) and ATF4‐overexpressing HCT116 cells (ATF4). I) Immunoblot showing the expression of Parkin in control HCT116 cells (Vector) and ATF4‐overexpressing HCT116 cells (ATF4). J,K) Control HCT116 cells (sg‐NC) and ATF4 knockout HCT116 cells (sg‐ATF4 1# or sg‐ATF4 2#) were transfected with Parkin‐promoter firefly luciferase reporter construct and a constitutive‐active Renilla luciferase reporter construct (pRL‐CMV), and then treated with 16 µm erastin for 22 h (J) or cystine deprivation for 16 h (K). Relative luciferase activity was measured using the Dual‐Luciferase Reporter Assay Kit (Promega E1980). L,M) Binding of ATF4 to a DNA fragment containing the CREB/ATF motif in the Parkin promoter. ChIP was performed on HCT116 (L) or MDA‐MB‐231 (M) cell lysate with antibodies against ATF4 and rabbit IgG as control. DNA fragments were amplified using the primers specific for CREB/ATF binding motif in the Parkin promoter region. The non‐coding region NC1 served as negative control. A, C, G, I, Data are representative of n =  3 biologically independent experiments. (B,D–F,H,J,K, Data are the mean ± s.d.; n =  3 biologically independent experiments. Statistical analysis was performed using an unpaired two‐tailed Student's t‐test.

To explore the mechanism by which mitophagy regulates cancer sensitivity to ferroptosis, we first analyzed the changes in mitophagy during the ferroptotic process in cancer cells. We treated cancer cells with erastin or RSL3 or IR to induce ferroptosis and simultaneously analyzed mitophagy during ferroptosis induction. We found that erastin or RSL3 or IR treatment increased mitophagy in a time‐ and concentration‐dependent manner in HCT116 and MDA‐MB‐231 cancer cells, as shown by decreased protein levels of the mitochondrial proteins TOM20 and TIMM23 and decreased mitochondrial DNA (mtDNA) (**Figure** **3**A,B; Figure S2A–H, Supporting Information). These results suggest that mitophagy is enhanced during the ferroptotic process in cancer cells, suggesting that enhanced mitophagy is involved in the regulation of cell fate during cancer cell ferroptosis.

We further explored the molecular mechanism by which mitophagy increases during the ferroptotic process. The expression levels of the mitophagy‐related genes Parkin, PINK1, FUNDC1, NIX, BNIP3, and BCL2L13 during ferroptosis were analyzed. Our results revealed that the ferroptosis inducer erastin strongly increased the mRNA and protein levels of Parkin without affecting other regulators of mitophagy in HCT116 and MDA‐MB‐231 cancer cells (Figure 3C,D; Figure S2I,J, Supporting Information). We used RSL3, another ferroptosis inducer, to verify the above conclusions. We found that RSL3 increased the mRNA and protein levels of Parkin in a concentration‐ and time‐dependent manner in HCT116 and MDA‐MB‐231 cancer cells (Figure S2K,L, Supporting Information). These results suggest that increased Parkin transcription during ferroptosis induction may be responsible for enhanced mitophagy in cancer cells. Subsequently, the transcription factor that mediates Parkin transcription was identified. The ferroptosis‐related transcription factors NFE2L2/NRF2, TP53, BACH1, SP1, ATF3, ATF4, YAP1, TAZ, TFEB, TFAP2C, HIF1A, EPAS1/HIF2A, JUN, HIC1, and HNF4A were individually knocked out in cancer cells, after which Parkin mRNA levels were analyzed (Figure 3E). The data revealed that knockout of ATF4 significantly attenuated the mRNA and protein levels of Parkin in cancer cells under physiological and erastin or RSL3 or cystine deprivation treatment conditions (Figure 3E–G; Figure S3A–J, Supporting Information). Correspondingly, ATF4 overexpression was sufficient to activate endogenous Parkin expression at both the mRNA and protein levels (Figure 3H,I; Figure S3K,L, Supporting Information). To determine whether ATF4 directly regulates Parkin gene transcription, we first analyzed the dependence of the transcriptional activity of a Parkin‐promoter‐luciferase reporter construct on ATF4 activity. Indeed, we observed that the Parkin promoter was fully responsive to the absence or presence of ATF4 under erastin or RSL3 or cystine deprivation treatment conditions (Figure 3J,K; Figure S3M–P, Supporting Information). Moreover, the transcription factor ATF4 usually has increased binding affinity for the CREB/ATF site located at the promoter of its target genes. Our analysis identified a potential CREB/ATF site on the Parkin promoter that is highly conserved across multiple species. Furthermore, a ChIP‒qPCR assay revealed that endogenous ATF4 readily bound to the DNA region containing the CREB/ATF motif in the promoter of the Parkin gene (Figure 3L,M). These results suggest that ATF4 is a transcription factor that mediates Parkin transcription.

Furthermore, we demonstrated that Parkin is transcriptionally upregulated by ATF4 to increase mitophagy and subsequently inhibit ferroptosis in cancer cells. Knockout of ATF4 inhibited ferroptosis inducer‐mediated mitophagy and enhanced erastin‐ or RSL3‐ or cystine deprivation‐induced ferroptosis and lipid peroxidation in cancer cells, and the overexpression of Parkin strongly reversed the effect of ATF4 knockout on cancer cells (Figure S4A–J, Supporting Information). These data suggested that the ATF4‐mediated expression of Parkin promoted mitophagy and then suppressed ferroptosis. Correspondingly, defective mitophagy is susceptible to ferroptosis in cancer cells. Mitochondrial reactive oxygen species (ROS), especially hydroxyl radicals, promote the production of ferroptosis‐related lipid peroxidation.^[^ ^23^, ^24^ ^]^ Our results revealed that mitochondrial ROS levels were strongly increased in erastin‐treated mitophagy‐deficient cancer cells compared with those in erastin‐treated wild‐type cancer cells (Figure S5A,D, Supporting Information). The mitochondrion‐targeted ROS scavenger MitoQ substantially decreased mitochondrial ROS, lipid peroxidation, and ferroptosis in mitophagy‐deficient cancer cells (Figure S5A–F, Supporting Information). Taken together, these results suggest that, during the ferroptotic process in cancer cells, enhanced mitophagy removes damaged mitochondria and reduces the production of mitochondrial ROS, which limits the generation of lipid peroxidation and ultimately weakens the occurrence of ferroptosis. In cancer cells with defective mitophagy, the large amount of mitochondrial ROS produced leads to the release of the restriction on the generation of lipid peroxidation, which makes cancer cells very sensitive to ferroptosis inducers.

### 2.3 The Lipid Peroxidation–ATF4–Parkin–Mitophagy Negative Feedback Pathway Limits the Generation of Lipid Peroxidation Products to Halt Ferroptosis in Cancer

**Figure 4:**
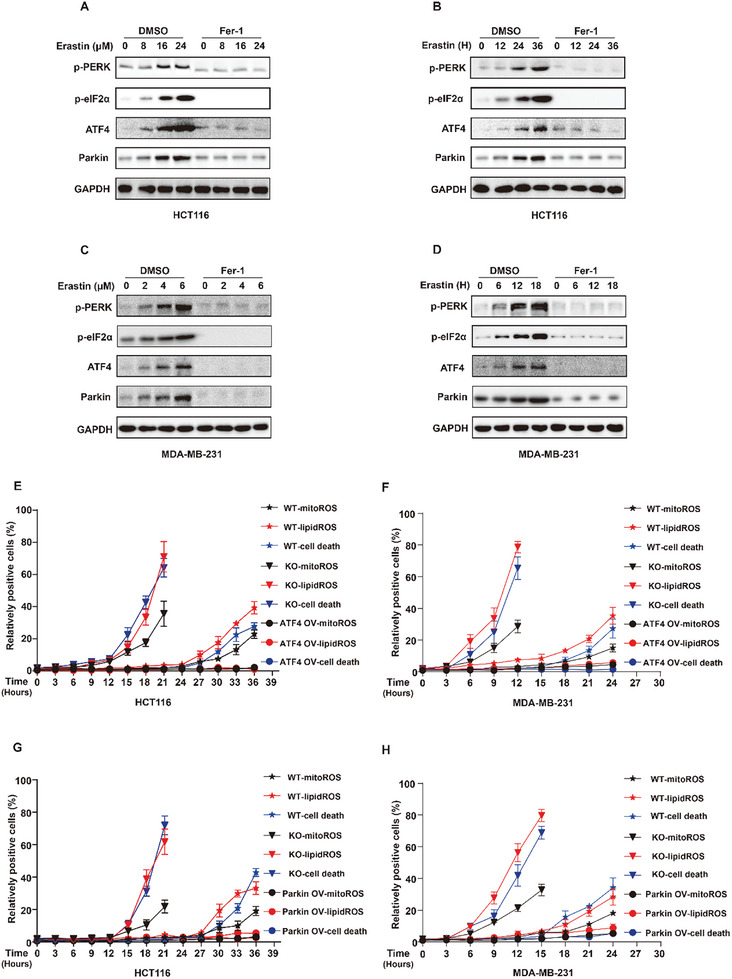
Lipid peroxidation‐ATF4‐Parkin‐mitophagy negative feedback pathway limits lipid peroxidation to halt ferroptosis in cancer. A–D) Immunoblot showing the expression of Parkin, ATF4, p‐PERK, eIF2α in HCT116 or MDA‐MB‐231 cells treated with erastin with or without 10 µm ferroptosis inhibitor ferrostatin‐1 (Fer‐1) at the indicated concentrations and times. A, time, 30 h. (B) concentration, 16 µm. C, time, 14 h. (D) concentration, 5 µm. (E–H) Mitochondrial ROS (mitoROS), lipid peroxidation (lipidROS), cell death measurement in HCT116 cells treated with 20 µm erastin for the indicated times (E,G) or in MDA‐MB‐231 cells treated with 3 µm erastin for the indicated times (F,H). (A–D) Data are representative of n =  3 biologically independent experiments.

We further explored the molecular mechanism of ATF4 activation in the ferroptotic process. ATF4 is an effector of endoplasmic reticulum stress. Our results showed that the ferroptosis inducers enhanced the protein level of ATF4. Therefore, we speculated that cancer cells might suffer from endoplasmic reticulum stress during ferroptosis induction. Western blot analysis revealed that erastin obviously activated endoplasmic reticulum stress, which manifested as the activation of the PERK/eIF2α/ATF4 signaling pathway in cancer cells (**Figure** **4**A–D). More importantly, the lipid peroxide scavenger ferrostatin‐1 (Fer‐1) strongly inhibited endoplasmic reticulum stress and reduced the expression of ATF4 and Parkin during ferroptosis induction (Figure 4A–D). These results indicated that, during ferroptosis induction, erastin treatment promoted ATF4 activation and then Parkin expression through lipid peroxidation‐mediated endoplasmic reticulum stress.

These results suggest that there may be a lipid peroxidation–ATF4–Parkin–mitophagy negative feedback pathway in cancer cells that resists ferroptosis by limiting the generation of lipid peroxidation products. Lipid peroxidation‐mediated endoplasmic reticulum stress induces the ATF4‒Parkin axis to promote mitophagy, limiting the generation of lipid peroxidation products and inhibiting ferroptosis by inhibiting the production and accumulation of mitochondrial ROS. To confirm the existence of this negative feedback pathway, we analyzed the effects of inhibiting or activating this pathway on mitochondrial ROS, lipid peroxidation, and ferroptosis in cancer cells at multiple consecutive time points during the ferroptotic process. Our results revealed that, compared with those in wild‐type cancer cells, mitochondrial ROS dramatically accumulated in the early stage of ferroptosis induction and gradually increased with erastin treatment in ATF4‐knockout HCT116 cancer cells (Figure 4E) or ATF4‐knockout MDA‐MB‐231 cancer cells (Figure 4F). Consistent with this result, lipid peroxidation and cell death in ATF4‐knockout cancer cells were significantly increased in the early stage of ferroptosis induction and gradually aggravated with the extension of erastin treatment compared with those in wild‐type cancer cells (Figure 4E,F). In contrast, mitochondrial ROS remained stable throughout the entire process of ferroptosis induction and did not significantly increase even at the late stage of ferroptosis induction in ATF4‐overexpressing cancer cells compared with wild‐type cancer cells (Figure 4E,F). Lipid peroxidation and cell death in cancer cells overexpressing ATF4 did not increase throughout the entire process of ferroptosis induction (Figure 4E,F). Consistently, we obtained similar conclusions by knocking out or overexpressing Parkin in cancer cells (Figure 4G,H). These results suggest that the lipid peroxidation–ATF4–mitophagy negative feedback pathway reduces the accumulation of mitochondrial ROS, which in turn limits the sharp increase in lipid peroxidation and maintains lipid peroxidation at a low level in cancer cells, thereby inhibiting the initiation of ferroptosis.

### 2.4 Mitophagy‐Deficient Tumors are Vulnerable to Ferroptosis

**Figure 5:**
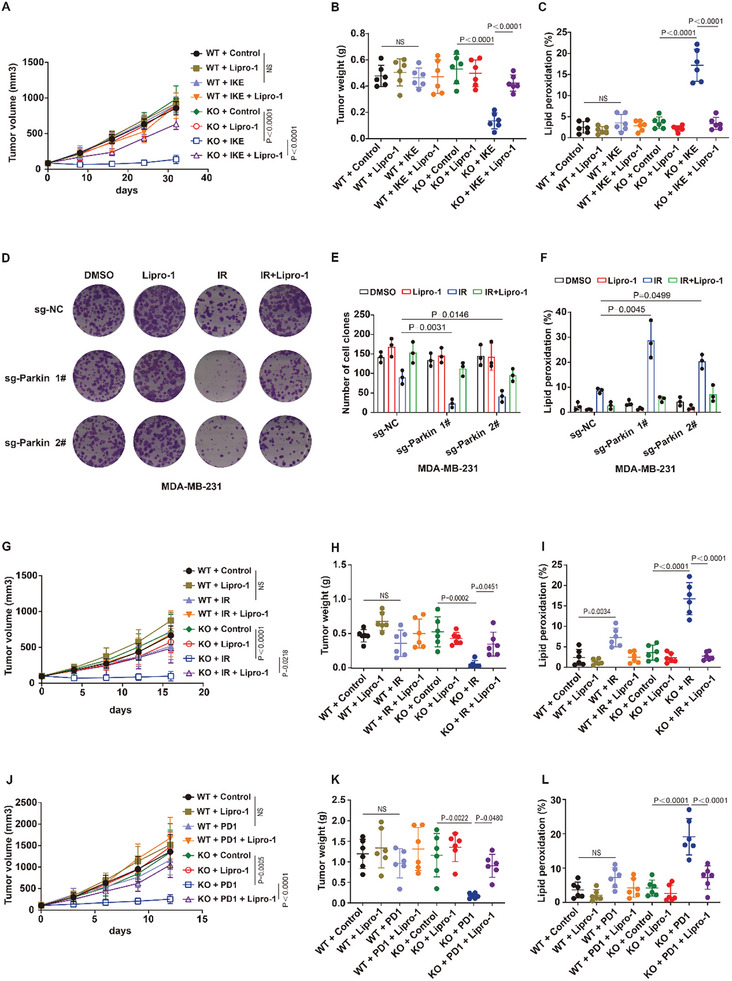
Mitophagy‐deficient tumor growth is vulnerable to ferroptosis. A,B) HCT116 cells were subcutaneously inoculated into BALB/c‐nu nude mice. Mice were randomly assigned to different treatment groups 10 days after tumor inoculation. Imidazole ketone erastin (IKE) was injected intraperitoneally into mice at a dose of 30 mg kg^−1^ every other day for 30 days. Lipro‐1 was administered three times before IKE followed by continued every other day administration at a dose of 15 mg kg^−1^ for 30 days. Tumor volumes (A) and tumor weights (B) of HCT116 xenograft tumors with the indicated treatments. C) Relative lipid peroxidation in tumor cells isolated from the indicated tumors. D–F) Cell clones (D,E) and lipid peroxidation (F) measurement in the indicated MDA‐MB‐231 cells treated with the indicated compounds or IR. Cell clones: IR, 2 Gy; lipro‐1, 5 µm. Lipid peroxidation: IR, 6 Gy; lipro‐1, 5 µm. G) MDA‐MB‐231 cells were subcutaneously inoculated into BALB/c‐nu nude mice. Mice were randomly assigned to different treatment groups 7 days after tumor inoculation. Tumors were irradiated with a JL Shepherd Mark I‐68A irradiator at a dose of 10 Gy. Lipro‐1 was administered three times before irradiation followed by continued once daily administration for 15 days. Volumes of MDA‐MB‐231 xenograft tumors with the indicated treatments at different time points (days). H) Tumor weights of MDA‐MB‐231 xenograft tumors with the indicated treatments after exposure to 10 Gy of IR. (I) Relative lipid peroxidation in tumor cells isolated from the indicated tumors. J) B16 cells were subcutaneously inoculated into C57 mice. On day 3, 100 µg anti‐PD1 (Bio X Cell), 15 mg kg^−1^ Lipro‐1 or both were administered intraperitoneally to each mouse. Antibodies were administered every 3 days and Lipro‐1 was administered three times before anti‐PD1 treatment followed by continued daily administration until the endpoint. Volumes of B16 tumors with the indicated treatments at different time points (days). K) Tumor weights of B16 tumors with the indicated treatments. (L) Relative lipid peroxidation in tumor cells isolated from the indicated tumors. A, G, J, Error bars are means ± SD, n = 6 independent repeats. P values were determined using 2‐way ANOVA. B‐F, H, I, K, L, Data are the mean ± s.d.; n =  6 biologically independent mice. Statistical analysis was performed using an unpaired two‐tailed Student's t‐test.

Mitophagy deficiency promotes the malignant progression of tumors. We speculate that highly malignant mitophagy‐deficient tumors lack the antiferroptosis mechanism mediated by the lipid peroxidation–ATF4–Parkin–mitophagy negative feedback pathway, which may increase the susceptibility of these tumors to ferroptosis. Wild‐type HCT116 or MDA‐MB‐231 cancer cells (Parkin‐WT, WT) and mitophagy‐deficient HCT116 or MDA‐MB‐231 cancer cells (Parkin‐knockout, KO) were subcutaneously inoculated into nude mice. The nude mice were subsequently treated with the ferroptosis inducer imidazole ketone erastin (IKE), Lipro‐1, or both. We found that treatment with IKE massively inhibited tumor growth and enhanced tumor lipid peroxidation in mice bearing Parkin‐knockout xenograft tumors compared with that in mice bearing Parkin‐WT, and Lipro‐1 treatment obviously inhibited the effect of IKE (**Figure** **5**A–C; Figure S6A–C, Supporting Information). These data suggest that mitophagy‐deficient tumors are sensitive to ferroptosis‐inducing therapy in vivo.

It has been reported that radiotherapy can exert its therapeutic effect by partially inducing ferroptosis. We constructed a radiotherapy model of tumors in vitro and in vivo. Our results revealed that radiotherapy strongly decreased the number of cell clones and increased lipid peroxidation in Parkin‐knockout breast cancer cells and colorectal cancer cells compared with those in wild‐type cancer cells (Figure 5D–F; Figure S6D–F, Supporting Information). Furthermore, we found that MDA‐MB‐231‐Parkin‐knockout xenograft tumors, but not MDA‐MB‐231‐WT xenograft tumors, were sensitive to radiotherapy, as shown by markedly elevated lipid peroxidation and stalled tumor growth (Figure 5G–I). Notably, Lipro‐1 treatment substantially reversed the radiotherapy‐mediated increase in lipid peroxidation and decreased tumor growth in Parkin‐knockout cancer cells or xenograft tumors (Figure 5D–I; Figure S6D–F, Supporting Information). In addition to radiotherapy, immunotherapy can also suppress tumors by inducing ferroptosis. We subcutaneously inoculated wild‐type B16 (B16‐WT) and mitophagy‐deficient B16 cancer cells (B16‐Parkin‐knockout) into immunocompetent C57 mice (Figure S6G, Supporting Information). After implantation, the mice were treated with an anti‐PD1 antibody, Lipro‐1, or both. Systemic PD1 antibody‐targeted treatment significantly elevated lipid peroxidation and inhibited tumor growth in immunocompetent C57 mice bearing B16‐Parkin‐knockout tumors compared with that in mice bearing B16‐WT tumors (Figure 5J–L). In addition, Lipro‐1 significantly inhibited the anti‐PD1‐mediated antitumor effect on Parkin‐knockout tumors (Figure 5J–L). Taken together, these results suggest that mitophagy‐deficient tumors are vulnerable to ferroptosis induced by IKE, radiotherapy, and immunotherapy.

### 2.5 Mitophagy‐Deficient Tumor Metastasis are Vulnerable to Ferroptosis

**Figure 6:**
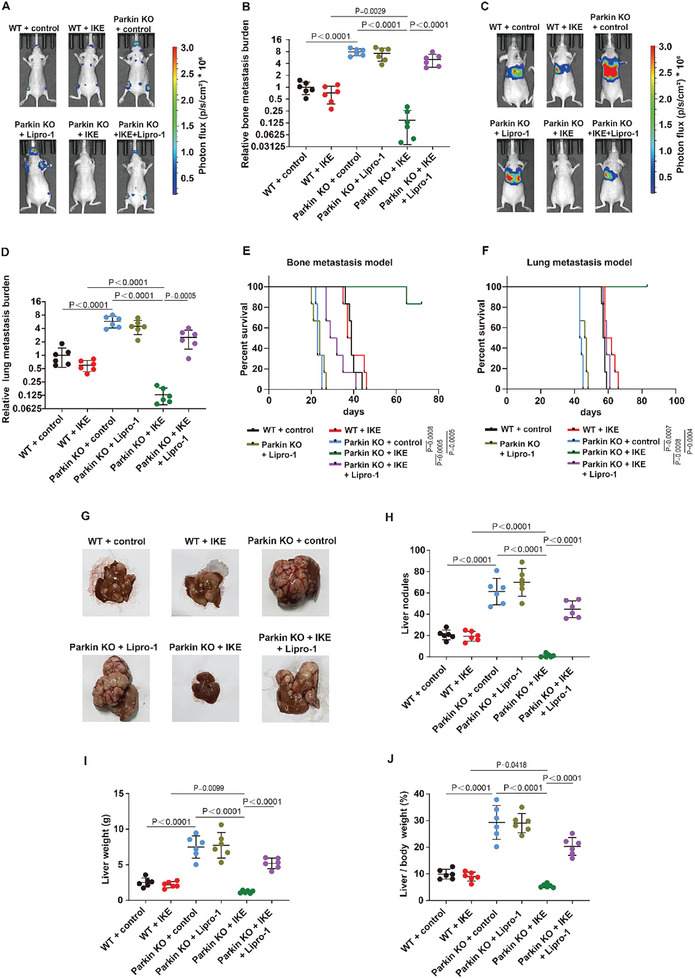
Mitophagy‐deficient tumor metastasis is vulnerable to ferroptosis. A,B) Mice were intracardiacally injected with wild‐type HCT116 or mitophagy‐deficient HCT116 cancer cells, then treated with control or IKE or Lipro‐1 or IKE and Lipro‐1. IKE was injected intraperitoneally into mice at a dose of 30 mg kg^−1^ once daily for 16 days starting from the day after cardiac injection of cancer cells. Lipro‐1 was administered three times before IKE treatment followed by continued daily administration at a dose of 15 mg kg^−1^ for 16 days starting from the day after cardiac injection of cancer cells. The relative bone metastasis burden of each group of mice was measured. C,D) Mice were tail‐vein injected with wild‐type HCT116 or mitophagy‐deficient HCT116 cancer cells, then treated with control or IKE or Lipro‐1 or IKE and Lipro‐1. IKE was injected intraperitoneally into mice at a dose of 30 mg kg^−1^ every other day for 36 days starting from the day after tail vein injection of cancer cells. Lipro‐1 was administered three times before IKE treatment followed by continued every other day administration at a dose of 15 mg kg^−1^ for 36 days starting from the day after tail vein injection of cancer cells. The relative lung metastasis burden of each group of mice was measured. E,F) Survival time of the indicated bone metastasis mice (E) or lung metastasis mice (F) treated with control or IKE or Lipro‐1 or IKE and Lipro‐1. G–J) Mice were injected intrasplenicly with wild‐type HCT116 or mitophagy‐deficient HCT116 cancer cells, then treated with control or IKE or Lipro‐1 or IKE and Lipro‐1. IKE was injected intraperitoneally into mice at a dose of 30 mg kg^−1^ every other day for 40 days starting from the day after the spleen injection of cancer cells. Lipro‐1 was administered three times before IKE treatment followed by continued every other day administration at a dose of 15 mg kg^−1^ for 40 days. The liver nodules (G,H), liver weight (I), liver/body weight ratio (J) in the indicated mice was measured. (A‐J) Data are the mean ± s.d.; n =  6 biologically independent mice. Statistical analysis was performed using an unpaired two‐tailed Student's t‐test.

Our studies and those of other researchers have shown that defects in mitophagy promote the distant metastasis of tumors, such as bone metastasis, lung metastasis, and liver metastasis. We further explored whether mitophagy‐deficient tumor metastasis is vulnerable to ferroptosis. Cancer cells stably expressing the luciferase reporter gene were injected into the left ventricle and into the tail vein to establish bone metastasis and lung metastasis models for colorectal cancer and breast cancer, respectively. The mice were treated with the solvent control or IKE as described above. We found that bone metastasis and lung metastasis were significantly greater in mice injected with mitophagy‐deficient cancer cells than in those injected with wild‐type cancer cells (**Figure** **6**A–D; Figure S7A–D, Supporting Information). Importantly, inducing ferroptosis via IKE strongly inhibited bone metastasis and lung metastasis in mice injected with mitophagy‐deficient tumor cells but not in mice injected with wild‐type tumor cells, and Lipro‐1 significantly inhibited the antimetastatic effect mediated by IKE in mitophagy‐deficient tumors (Figure 6A–D; Figure S7A–D, Supporting Information). We next determined whether IKE treatment affects the survival of mice injected with mitophagy‐deficient tumor cells. The results revealed that IKE treatment significantly increased the median overall survival of lung metastasis model mice and bone metastasis model mice and that Lipro‐1 treatment substantially shortened the survival time of IKE‐treated mice bearing mitophagy‐deficient tumors (Figure 6E,F; Figure S7E,F, Supporting Information), further confirming the potent antimetastatic activity of ferroptosis in mitophagy‐deficient cancer. We used intrasplenic injection of wild‐type or mitophagy‐deficient cancer cells to induce liver metastasis in both breast cancer and colorectal cancer. The mice in the wild‐type group or mitophagy‐deficient group were then treated with the solvent control or IKE. Our results revealed that, compared with those injected with wild‐type cancer cells, mice injected with mitophagy‐deficient cancer cells had significantly increased liver metastasis, as demonstrated by increased intrahepatic tumor nodules, liver weights, and liver weight‐to‐body weight ratios (Figure 6G–J; Figure S7G–J, Supporting Information). Similar to the results of the abovementioned lung metastasis and bone metastasis models, IKE could strongly inhibit liver metastasis of mitophagy‐deficient tumor cells but not wild‐type tumor cells. IKE treatment sharply reduced the number of intrahepatic tumor nodules, liver weight, and the liver weight‐to‐body weight ratio in mice injected with mitophagy‐deficient cancer cells (Figure 6G–J; Figure S7G–J, Supporting Information). Lipro‐1 significantly inhibited the antimetastatic effect of IKE in mitophagy‐deficient tumors (Figure 6G–J; Figure S7G–J, Supporting Information). Collectively, these results indicate that mitophagy‐deficient tumor metastasis is vulnerable to ferroptosis.

## 3 Discussion

**Figure 7:**
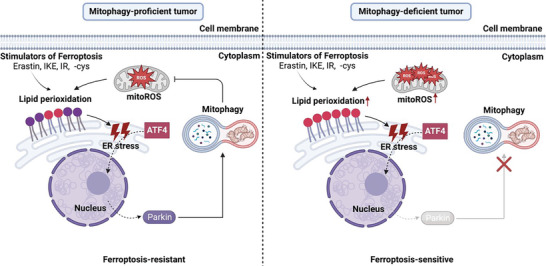
Proposed model of the susceptibility of mitophagy‐deficient tumors to ferroptosis induction. (Left), During the ferroptotic process in cancer cells, lipid peroxidation‐mediated endoplasmic reticulum stress (ER stress) activates ATF4, and then ATF4 transcriptionally upregulates Parkin to promote mitophagy. Mitophagy reduces the production and accumulation of mitochondrial ROS (mitoROS), thereby limiting the generation of lipid peroxidation and the occurrence of ferroptosis. (Right), In cancer cells with defective mitophagy, the large amount of mitochondrial ROS (mitoROS) produced leads to the release of the restriction on the generation of lipid peroxidation, which makes the cancer cells or tumors susceptible to ferroptosis, and unleashes the generation of lipid peroxidation and potent ferroptotic cell death induced by erastin, IKE, cysteine deprivation (‐cys), radiotherapy (IR) and immunotherapy.

Inducing ferroptosis in cancer cells is a potential antitumor strategy. However, which specific types of cancers are sensitive to ferroptosis‐inducing agents? Here, by performing patient‐derived organoid screening models of colorectal cancer, we revealed that tumors deficient in mitophagy are hypersensitive to ferroptosis‐inducing therapies and that inducing ferroptosis selectively inhibits the growth and distant metastasis of mitophagy‐deficient tumors in vivo. Mechanistically, we found that cancer cells activated mitophagy through the lipid peroxidation–ATF4–Parkin pathway to limit the generation of lipid peroxidation products, thereby inhibiting the occurrence of ferroptosis. Mitophagy is one of the intrinsic anti‐ferroptosis mechanisms of cancer cells, and the lack of this anti‐ferroptosis mechanism in highly malignant mitophagy‐deficient tumors makes these tumors vulnerable to ferroptosis (**Figure** **7**).

Ferroptosis is thought to be the next breakthrough in cancer therapy. It has been reported that cancer cells resistant to chemotherapy and targeted therapy are particularly sensitive to ferroptosis‐inducing agents,^[^ ^25^ ^]^ suggesting that the induction of ferroptosis can overcome tumor resistance to proapoptotic drugs. An increase in ACSL4 expression and ROS levels in radiotherapy‐treated cancer cells promotes ferroptosis.^[^ ^26^ ^]^ Inhibition of ferroptosis impairs the efficacy of radiotherapy,^[^ ^26^ ^]^ suggesting that radiotherapy‐mediated ferroptosis promotes the efficacy of radiotherapy. During immunotherapy, activated CD8^+^ T cells promote tumor cell ferroptosis by secreting IFNγ to reduce the expression of SLC7A11 and increase the expression of ACSL4 in tumor cells.^[^ ^27^, ^28^ ^]^ Lipro‐1, an inhibitor of ferroptosis, significantly inhibited the efficacy of immunotherapy,^[^ ^27^, ^28^ ^]^ suggesting that ferroptosis is one of the mechanisms by which immunotherapy suppresses tumors. These studies show that the induction of ferroptosis is a promising antitumor strategy. The sensitivity of tumors to ferroptosis varies widely and is highly heterogeneous across different genetic backgrounds. The identification of tumor types that are vulnerable to ferroptosis will facilitate the successful application of ferroptosis in cancer therapy. Here, we established patient‐derived organoids of colorectal cancer patients for screening ferroptosis‐sensitive tumors, providing a paradigm for identifying which patient‐derived tumors are sensitive to ferroptosis‐inducing therapies. This provides a theoretical and methodological basis for the future clinical selection of tumor patients who are sensitive to ferroptosis‐inducing therapy. We conclude that mitophagy‐deficient tumors are vulnerable to ferroptosis induction, which may lead to the development of new therapeutic strategies for patients with tumors characterized by defective mitophagy. We also identified the transcription and protein levels of the mitophagy genes such as PINK1 or Parkin as potential molecular markers for predicting ferroptosis sensitivity in colorectal cancer organoids and multiple cancer cell lines.

This finding has excellent clinical translational implications. Exploiting the pathological mechanisms of diseases will help us discover new targets and develop new therapeutic drugs and treatments.^[^ ^4^, ^29^, ^30^, ^31^, ^32^, ^33^ ^]^ Mitochondria are the energy factories of cells and are crucial to determining the fate of cells. Abnormal regulation of mitochondria promotes the occurrence and progression of many diseases, and maintaining mitochondrial homeostasis is a therapeutic strategy for these diseases.^[^ ^29^, ^34^, ^35^, ^36^, ^37^, ^38^ ^]^ Autophagy defects are also closely related to the occurrence and development of various diseases.^[^ ^39^, ^40^, ^41^, ^42^, ^43^, ^44^ ^]^ Our previous studies have shown that tumors with defective mitophagy are prone to distant metastasis due to high levels of mitochondrial ROS.^[^ ^10^ ^]^ Here, we found that this high level of mitochondrial ROS also made such tumors with defective mitophagy more sensitive to ferroptosis inducers. These results suggest that targeted induction of ferroptosis can selectively kill malignant tumors with defective mitophagy. In future clinical practice, the patients with tumors with defective mitophagy can be screened by detecting the expression of mitophagy genes such as Parkin or PINK1 in the patient's tumor tissue, and then the patients are treated with ferroptosis‐inducing therapy, which will greatly promote the therapeutic efficacy of such patients. However, specific ferroptosis inducers have not yet been approved for clinical use, so the above clinical tumor treatment plans cannot be carried out in patients at present.

Mitochondria are closely related to cell death.^[^ ^45^ ^]^ The release of mitochondrial cytochrome C (Cytochrome‐c) mediates the occurrence of apoptosis by activating caspase‐9.^[^ ^46^ ^]^ The transport of Smac/DIABLO from the mitochondria to the cytoplasm promotes apoptosis by inhibiting the IAP.^[^ ^47^ ^]^ Recent studies suggest that mitochondria are also closely related to ferroptosis. Mitochondrial morphological changes, such as mitochondrial fragmentation and crista enlargement, are among the hallmarks of ferroptosis.^[^ ^1^, ^18^, ^20^ ^]^ However, the regulatory effect of mitophagy, an important aspect of mitochondrial homeostasis, on ferroptosis in cancer cells remains unclear. Our study revealed that ferroptosis inducers can enhance cancer cell mitophagy in a time‐ and concentration‐dependent manner. Mechanistically, lipid peroxidation triggers mitophagy by activating the ATF4–Parkin axis. Mitophagy clears damaged mitochondria and mitochondrial ROS, thereby attenuating the amplification of lipid peroxidation and inhibiting ferroptosis. Thus, we discovered a novel ferroptosis resistance pathway. Lipid peroxidation–ATF4–Parkin limits the production of lipid peroxidation products by activating mitophagy, and this negative feedback regulatory pathway of lipid peroxidation plays an important role in the process of cancer cell resistance to ferroptosis. Inhibition or deletion of the lipid peroxidation–ATF4–Parkin negative feedback pathway has strong clinical translational value. Highly malignant mitophagy‐deficient tumors lack this negative feedback antiferroptosis mechanism, making tumors susceptible to ferroptosis, and unleash potent ferroptotic cell death induced by erastin, RSL3, cysteine deprivation, radiotherapy, and immunotherapy. More importantly, ferroptosis‐inducing therapies selectively inhibit the growth and distant metastasis of mitophagy‐deficient tumors.

## 4 Experimental Section

### Cells

Human colorectal cancer HCT116 (CCL‐247), human breast cancer MDA‐MB‐231 (CRM‐HTB‐26), murine B16 (CRL‐6475) cell lines were obtained from the American Type Culture Collection. The cancer cell lines were cultured in DMEM with 10% fetal bovine serum (FBS) at 37 °C in an incubator with a humidified atmosphere of 20% O2 and 5% CO2.

### Animals

Irradiation and cell line‐derived xenograft models used athymic nude mice aged 4–6 weeks. For the B16 tumor model, 2.0 ×1 0^5^ B16 cells were subcutaneously injected into the left flank of C57 mice. Female athymic nude mice (BALB/c‐nu) and C57 mice (C57BL/6) were purchased from Gempharmatech‐GD and housed in a specific pathogen‐free animal facility. All animal procedures were performed in accordance with institutional guidelines and with approval from the Institutional Animal Care and Use Committee (IACUC) of Sun Yat‐sen University Cancer Center.

### Construction of Colorectal Cancer Organoids

*Sample Collection*: Colorectal cancer samples were obtained from the Kiang Wu Hospital, Macau. Prior patient written consents were acquired from donors with informing the use for organoid culture, drug testing, genomics sequencing, publication, and other associated scientific studies. This study was assessed and approved by the ethics committees of the University of Macau, Kiang Wu Hospital.

*Tissue Dissociation*: The fresh tumor tissues were primarily minced into 1–3 mm 3 pieces. Two random pieces were used for DNA separation and formalin fixation. The remaining portions were added with digestion buffer. The tissues were digested at 37 °C for ≈1 h with gentle shaking until the visible pieces disappeared. Dissociated cell clusters were centrifuged at 2000 rpm for 3 min, washed with DMEM supplemented with 10% FBS, and spun down again at 2000 rpm for 3 min. After treating with RBC lysis buffer (eBiosciences) for 3 min, the remaining tumor cells were collected for organoid culture and cryopreservation.

### Organoid Culture

Dissociated tumor cells were resuspended in Matrigel solution, then seeded in prewarmed‐well culture plates at 31 µL per drop. Once cell Matrigel drops were solidified at 37 °C, 2.5 mL/well organoid culture medium was added into initiate continuous culture. Medium were refreshed every 2–3 days and passage developed every 5–10 days depending on organoid density and size.

### Drug Screening

Imidazole ketone erastin (IKE) was dissolved in 5% DMSO/95% Hank's Balanced Salt Solution (HBSS) at the concentration of 20 mm, then diluted in sterile PBS and arrayed in 384‐well plates at 5‐point serial dilutions (2.5‐40 µm, as 10× working dilution). First, organoids were enzymatically dissociated into single cells, then diluted with the medium for drug screening (organoid culture medium removed Y‐27632, SB202190, and A83‐01; supplemented with 2.5% Matrigel). Cells were seeded in type‐I collagen gel precoated 384‐well plate at a density of ≈3000 cells per well. The third day, drugs were added into each well with 5‐point dilutions, and cells were incubated with drugs for 4 days. Three technical replicates of each drug were tested on three plates. To examine organoid viability, quantifying ATP content in each well as the proxy for metabolically viable cells using the CellTiter‐LumiTM Plus assay (Beyotime) was applied. Luminescence readout from drug‐treated wells were normalized against control wells and expressed as percentage cell viability.

### Plasmid Constructs

Plasmids containing the Parkin gene and PINK1 gene and ATF4 gene were purchased from Sino Biological, and PCDH‐NEO or pcDNA3.1 was used as the carrier vector for genes. These genes were integrated into the pCDH‐Neo or pcDNA3.1 destination vector using Gateway cloning. CRISPR–Cas9 technology was used to knock out Parkin, PINK1, and ATF4. Each sgRNA was cloned into the empty backbone of lenti‐CRISPR v2.

### Cell Death Assay

Cell death was analyzed by propidium iodide (PI) staining. Cancer cells were seeded in 6‐well plates at a density of 3 × 10^5^ cells per well or 12‐well plates at a density of 1 × 10^5^ cells per well. The next day, cancer cells were treated with erastin or cultured in cystine‐free medium, and then viable cells and dead cells were collected, stained with 5 µg mL‐1 PI, and analyzed by flow cytometry. PI‐positive cells were considered dead cells. At least 5000 cells were analyzed in each group, and all experiments were repeated at least three times.

### Lipid Peroxidation Assay

Cells were seeded in 6‐well plates at a density of 3 × 10^5^ per well or 12‐well plates at a density of 1 × 10^5^ per well. On the second day, the cells were treated with erastin or cultured in cystine‐free medium. Then, the cells were stained with 5 µm C11‐BODIPY 581/591 at 37 °C for 30 min and analyzed by flow cytometry. For BODIPY 581/591 C11 staining, the signals from both nonoxidized C11 (PE channel) and oxidized C11 (FITC channel) were monitored. The ratio of the MFI of FITC to the MFI of PE was calculated for each sample. Cells undergoing lipid peroxidation were defined as those having a high FITC/PE fluorescence ratio. The boundaries that defined what constituted an increased FITC/PE fluorescence ratio were set based on untreated cancer cells, a condition that represents cells with little or no lipid peroxidation. Lipid ROS‐positive cells were defined as cells with a FITC/PE fluorescence ratio greater than 98% of the untreated cells. At least 5000 cells were analyzed in each group, and all experiments were repeated at least three times.

### Immunoblotting

For immunoblotting, the protein concentration was determined after the cells were lysed in RIPA buffer. 30 micrograms of total protein were separated by SDS–PAGE and transferred to a PVDF membrane. Antibodies to the following were used: Parkin (CST, 2132S; 1:1000), PINK1 (CST, 6946S; 1:1000), ATF4 (CST, 11815S; 1:2000), TOM20 (Santa Cruz, sc‐17764; 1:1000), TIM23 (CST, 34822S; 1:2000), and Phospho‐PERK (CST, 3179S; 1:1000).

### Mitochondrial ROS Assay

For measurement of mitochondrial ROS, cells were stained for 15 min at 37 °C with 5 µm MitoPY1 (R&D Systems, 4428), and analyzed by flow cytometry. Flow cytometry analysis was conducted on Gallios (Beckman) and the data was analyzed using FlowJo software according to manufacturers’ instructions.

### Dual‐Luciferase Report Assay

MDA‐MB‐231‐WT or MDA‐MB‐231‐sg‐ATF4 or HCT116‐WT or HCT116‐sg‐ATF4 cells were seeded into 24‐well plates, on the second day, the cells were treated with erastin or cultured in cystine‐free medium. Then, pGL4.10‐Parkin and pRL‐CMV were transfected together into cells at a 10:1 mass ratio, and medium was changed after 4 h. The cells were washed with PBS twice, and Firefly luminescence and Renilla luminescence were measured using the Dual‐Luciferase report Assay Kit (Promega E1980) and a bioluminescence plate reader (Berthold Centro LB 960).

### ChIP qPCR

MDA‐MB‐231 or HCT116 cancer cells were treated with erastin, and then the cells were fixed in 1% formaldehyde for 10 min at room temperature, subsequently harvested and lysed in ChIP lysis buffer (1% SDS, 5 mm EDTA, 50 mm Tris‐HCl, pH 8.1) supplemented with protease inhibitors. Chromatin was fragmented using a sonicator (Branson), then diluted and immunoprecipitated with normal IgG or ATF4 antibodies overnight at 4 °C. On the second day, the protein G‐Sepharose beads were added and incubated at 4 °C for 1 h. The beads were washed sequentially with TSE I (0.1% SDS, 1% Triton X‐100, 2 mm EDTA, 20 mm Tris‐HCl, pH 8.1, 150 mm NaCl), TSE II (0.1% SDS, 1% Triton X‐100, 2 mm EDTA, 20 mm Tris‐HCl, pH 8.1, 500 mm NaCl), and buffer III (0.25 m LiCl, 1% NP40, 1% deoxycholate, 1 mm EDTA, 10 mm Tris‐HCl, pH 8.1) for 15 min each and twice with TE buffer. The chromatin was released from the beads using elution buffer (1% SDS with 0.1 m NaHCO3) and de‐cross‐linked by heating at 65 °C overnight. Input and IP samples were purified with QIAquick Gel Extraction kit (Qiagen, #28 704), and eluted DNA samples were analyzed by quantitative PCR. Fold enrichments for specific Parkin promoter regions were calculated by IP over input samples and normalized to isotype‐specific IgG as the negative control.

### Irradiation and Clonogenic Survival Assay

To determine the effect of mitophagy on IR‐induced ferroptosis, cancer cells were seeded in 6‐well plates at a density of 800 cells per well. The next day, the cells were pretreated with erastin or Lipro‐1 for 6 h, irradiated at doses from 2 Gy and 250 MU/min, and cultured in normal medium. Fresh medium containing different compounds was added to the plates every 2 days. After incubation for 2 weeks, the cells were stained with 0.5% crystal violet dissolved in 20% methanol. The colonies in each well were counted visually.

### HCT116‐ and MDA‐MB‐231‐Derived Xenograft Model

The study complied with all relevant ethical regulations. All procedures involving mice and experimental protocols were approved by the Institutional Animal Care and Use Committee (IACUC) of Sun Yat‐sen University Cancer Center. Female 4‐ to 6‐week‐old athymic nude mice were obtained from Gempharmatech‐GD. All mice were kept under specific‐pathogen free conditions in the animal facility of Sun Yat‐sen University Cancer Center. HCT116 or HCT116‐Parkin‐KO or MDA‐MB‐231 or MDA‐MB‐231‐Parkin‐KO cancer cell lines were suspended and counted in 1×DMEM, and the cancer cells were subcutaneously inoculated into female nude mice. When the tumors reached 50–150 mm^3^, the mice were randomly assigned to different treatment groups. Tumors were irradiated with a JL Shepherd Mark I‐68A irradiator at a dose of 10 Gy. Imidazole ketone erastin (IKE) was dissolved in 5% DMSO/95% Hank's Balanced Salt Solution (HBSS) at pH 4, and injected intraperitoneally into mice at a dose of 30 mg kg^−1^ every other day for 30 days in HCT116 colorectal cancer xenografts. In MDA‐MB‐231 breast cancer xenografts, 30 mg kg^−1^ IKE was injected intraperitoneally into mice once daily for 15 days. Lipro‐1 diluted in PBS was intraperitoneally injected at a dose of 15 mg kg^−1^. Lipro‐1 was administered three times before IKE followed by continued every other day administration for 30 days in HCT116 colorectal cancer xenografts, as indicated in the corresponding figures. In MDA‐MB‐231 breast cancer xenografts, Lipro‐1 was administered three times before irradiation or IKE followed by continued once daily administration for 15 days. MDA‐MB‐231 or HCT116 xenograft tumor volume was measured every 4 days or 8 days until the endpoint and calculated according to the equation volume = length×width^2^×1/2. For lipid peroxidation analysis of tumors in mice, tumors were isolated into single cells and stained with BODIPY 581/591 C11 and analyzed lipid peroxidation by flow cytometry. The y‐axis represents lipid peroxidation‐positive cells.

### B16 Tumor Model

To explore the impact of Parkin knockout‐mediated mitophagy‐deficient on the efficacy of immunotherapy, 2 × 10^5^ B16‐WT or B16‐Parkin knockout cells were subcutaneously inoculated into C57 mice. On day 3, 100 µg anti‐PD1 (Bio X Cell), 15 mg kg^−1^ Lipro‐1 or both were administered intraperitoneally to each mouse. Antibodies were administered every 3 days and Lipro‐1 was administered three times before anti‐PD1 treatment followed by continued daily administration until the endpoint. Tumor diameters were measured using calipers. After the mice were killed, the tumor tissues were excised and weighed. For lipid peroxidation analysis of tumors in mice, tumors were isolated into single cells and stained with BODIPY 581/591 C11 and analyzed lipid peroxidation by flow cytometry. The y‐axis represents lipid peroxidation‐positive cells.

### Colorectal Cancer and Breast Cancer Bone Metastasis and Lung Metastasis Models

Female BALB/c nude mice were obtained from Gempharmatech‐GD. All procedures involving mice and experimental protocols were approved by the Institutional Animal Care and Use Committee (IACUC) of Sun Yat‐sen University Cancer Center. For bone metastasis studies, 2 × 10^5^ luciferase‐tagged HCT116‐WT or HCT116‐Parkin‐knockout or MDA‐MB‐231‐WT or MDA‐MB‐231‐Parkin‐knockout cancer cells were injected into the left cardiac ventricle of female nude mice. For lung metastasis studies, 2×10^6^ luciferase‐labeled HCT116‐WT or HCT116‐Parkin‐knockout or MDA‐MB‐231‐WT or MDA‐MB‐231‐Parkin‐knockout cancer cells were injected into the tail vein of female nude mice.14 days after cardiac injection or 36 days after tail vein injection, the development of bone or lung metastases was monitored by measuring the photon flux of BLI signals in the forelimbs or lungs of mice after intraperitoneal injection of 100 mg kg^−1^ D‐luciferin (PerkinElmer, 122799). Bioluminescence images were acquired with the IVIS Imaging System (Xenogen). BLI signal data were acquired after background subtraction. Data were normalized to the signal obtained immediately after xenografting (day 0). To determine the therapeutic effect of Imidazole ketone erastin (IKE) on metastasis, mice were randomly assigned to six groups. For bone metastasis experiments, IKE was dissolved in 5% DMSO/95% Hank's Balanced Salt Solution (HBSS) at pH 4, and injected intraperitoneally into mice at a dose of 30 mg kg^−1^ once daily for 16 days starting from the day after cardiac injection of cancer cells. Lipro‐1 diluted in PBS was intraperitoneally injected daily at a dose of 15 mg kg^−1^. Lipro‐1 was administered three times before IKE treatment followed by continued daily administration for 16 days starting from the day after cardiac injection of cancer cells. For lung metastasis experiments, IKE was dissolved in 5% DMSO/95% Hank's Balanced Salt Solution (HBSS) at pH 4, and injected intraperitoneally into mice at a dose of 30 mg kg^−1^ every other day for 36 days starting from the day after tail vein injection of cancer cells. Lipro‐1 diluted in PBS was intraperitoneally injected daily at a dose of 15 mg kg^−1^. Lipro‐1 was administered three times before IKE treatment followed by continued every other day administration for 36 days starting from the day after tail vein injection of cancer cells.

### Colorectal Cancer and Breast Cancer Liver Metastasis Model

Female BALB/c nude mice were obtained from Gempharmatech‐GD. All procedures involving mice and experimental protocols were approved by the Institutional Animal Care and Use Committee (IACUC) of Sun Yat‐sen University Cancer Center. 1 × 10^6^ HCT116‐WT or HCT116‐Parkin‐knockout or MDA‐MB‐231‐WT or MDA‐MB‐231‐Parkin knockout cancer cells were injected into the spleen of female nude mice. To determine the therapeutic effect of Imidazole ketone erastin (IKE) on colorectal cancer and breast cancer liver metastasis, mice were randomly assigned to six groups. IKE was dissolved in 5% DMSO/95% Hank's Balanced Salt Solution (HBSS) at pH 4, and injected intraperitoneally into mice at a dose of 30 mg kg^−1^ every other day for 40 days starting from the day after the spleen injection of cancer cells. Lipro‐1 diluted in PBS was intraperitoneally injected every other day at a dose of 15 mg kg^−1^. Lipro‐1 was administered three times before IKE treatment followed by continued every other day administration for 40 days. The mice were sacrificed 42 days after the spleen was injected with cancer cells, and the liver tissues were separated, weighed, and photographed.

### Statistical analysis

Statistical analyses were conducted using GraphPad Prism 8.0.1. (GraphPad, La Jolla, CA, USA) and SPSS 20 software. The results are presented as the mean ± SD of three or six biologically independent experiments or samples. The results were analyzed by unpaired Student's *t*‐test, one‐way or two‐way ANOVA with Dunnett's multiple comparisons test, one‐way or two‐way ANOVA with Tukey's multiple comparisons test, or Wilcoxon matched‐pairs signed rank test using GraphPad Prism. All statistical tests were two‐sided, and a P value less than 0.05 was considered significant.

## Conflict of Interest

The authors declare no conflict of interest.

## Author Contributions

S.L., J.‐H.C., and L.‐C.L. contributed equally to this work. H.‐L.Z. and X.‐F.Z. conceived the idea. H.‐L.Z., S.L., J.‐H.C., and L.‐C.L. performed most experiments. J.‐H.C. and C.‐X.D. performed the construction of colorectal cancer organoids. Z.‐P.Y., B.‐X.H., J.‐H.T., Y.‐H.C., and G.‐K.F. performed the animal experiments. H.‐L.Z., R.D. and X.‐F.Z. wrote the manuscript. J.‐N.L. edited the revised manuscript. All co‐authors have seen and approved the manuscript.

## Supporting information



Supporting Information

## Data Availability

The data that support the findings of this study are available from the corresponding author upon reasonable request.
