# Supplementary material for: Susceptibility of Mitophagy‐Deficient Tumors to Ferroptosis Induction by Relieving the Suppression of Lipid Peroxidation
Source: Adv Sci (Weinh). 2024 Dec 16;12(6):2412593. doi: 10.1002/advs.202412593 (PMC11809388; doi:10.1002/advs.202412593)
Supplement: Supplementary file 1 — Supporting Information [file ADVS-12-2412593-s001.docx]

**Susceptibility of mitophagy-deficient tumors to ferroptosis induction**

**by relieving the suppression of lipid peroxidation**

*Shan Liu^1,2,3,7^, Jing-Hong Chen^4,5,7^, Li-Chao Li^1,7^, Zhi-Peng Ye^1^, Jian-Nan Liu^6^, Yu-Hong Chen^1^, Bing-Xin Hu^1^, Jia-Hong Tang^1^, Gong-Kan Feng^1^**, Zhi-Ming Li^1,3^, Chu-Xia Deng^5^, Rong Deng^1^, Xiao-Feng Zhu^1^*, Hai-Liang Zhang^1^**

^1^ State Key Laboratory of Oncology in South China, Guangdong Provincial Clinical

Research Center for Cancer, Guangdong Key Laboratory of Nasopharyngeal Carcinoma Diagnosis and Therapy, Sun Yat-sen University Cancer Center, Guangzhou, China.

^2^ Department of Medical Oncology, The Seventh Affiliated Hospital, Sun Yat-sen University, Shenzhen 518107, China.

^3^ Department of Medical Oncology, Sun Yat-sen University Cancer Center, Guangzhou, Guangdong, China

^4^ Guangzhou Municipal and Guangdong Provincial Key Laboratory of Protein Modification and Degradation, School of Basic Medical Sciences, Guangzhou Medical University, Guangzhou, China.

^5^ Faculty of Health Sciences, University of Macau, Macau SAR, China.

^6^ Department of Oncology, The Affiliated Yantai Yuhuangding Hospital of Qingdao University, Yantai, China.

^7^ These authors contributed equally: Shan Liu, Jing-Hong Chen, Li-Chao Li.

* Correspondence authors:

Hai-Liang Zhang, State Key Laboratory of Oncology in South China, Sun Yat-sen University Cancer Center, 651 Dongfeng Road East, Guangzhou, 510060, China, E-mail: zhanghl@sysucc.org.cn

Xiao-Feng Zhu, State Key Laboratory of Oncology in South China, Sun Yat-sen University Cancer Center, 651 Dongfeng Road East, Guangzhou, 510060, China, E-mail: zhuxfeng@mail.sysu.edu.cn

**Figure. S1.**


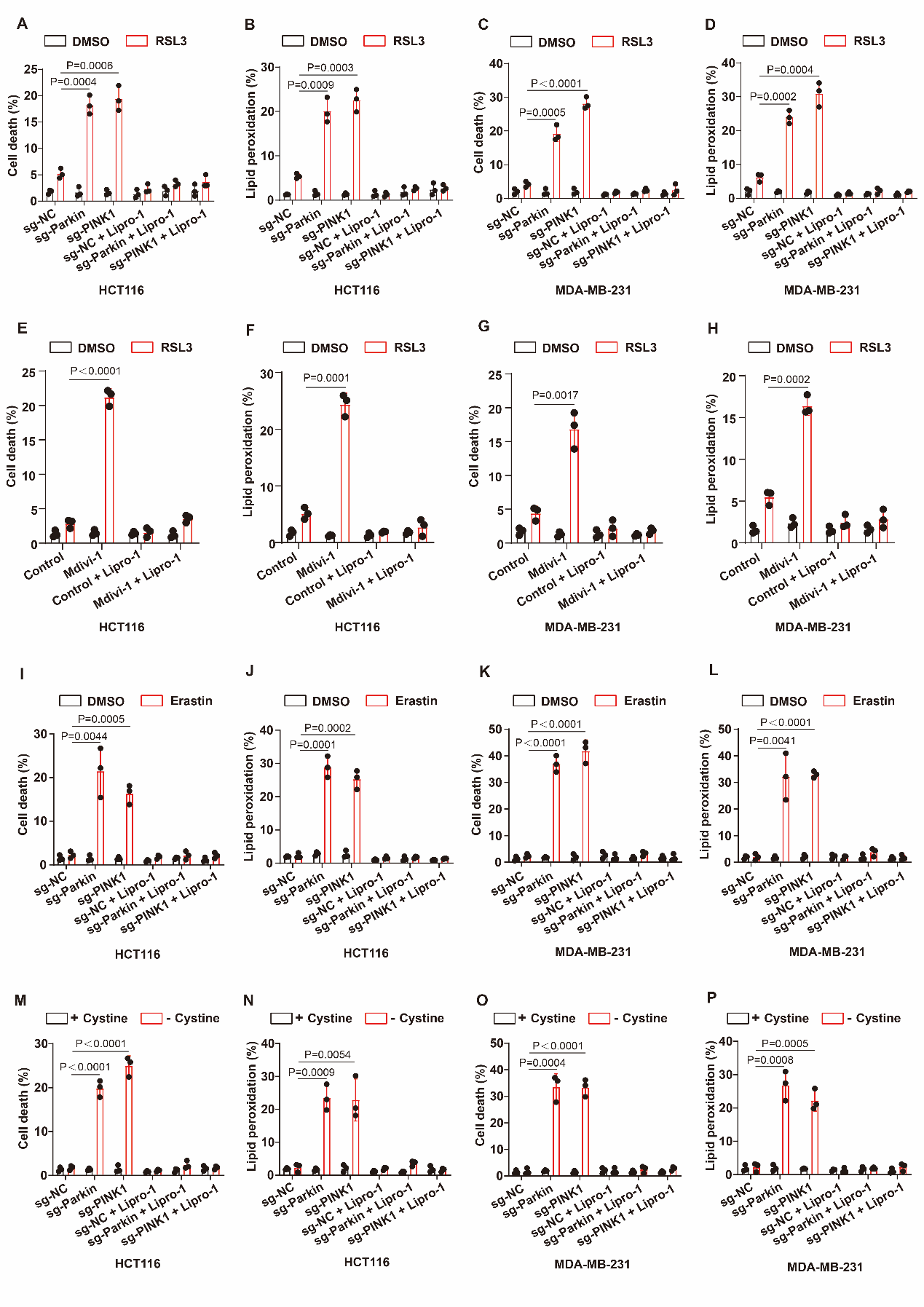


**Figure. S1. Mitophagy-deficient colorectal cancer cells and breast cancer cells are sensitive to ferroptosis inducers. (A, B)** Cell death **(A)** and lipid peroxidation **(B)** measurement in the indicated HCT116 cells treated with 6 μM RSL3 for 18 h with or without 10 μM Lipro-1. **(C, D)** Cell death **(C)** and lipid peroxidation **(D)** measurement in the indicated MDA-MB-231 cells treated with 1 μM RSL3 for 12 h with or without 10 μM Lipro-1. **(E, F)** Cell death **(E)** and lipid peroxidation **(F)** measurement in the indicated HCT116 cells treated with 6 μM RSL3 for 18 h with or without 10 μM Mdivi-1 or 10 μM Lipro-1. **(G, H)** Cell death **(G)** and lipid peroxidation **(H)** measurement in the indicated MDA-MB-231 cells treated with 1 μM RSL3 for 12 h with or without 10 μM Mdivi-1 or 10 μM Lipro-1. **(I, J)** Cell death **(I)** and lipid peroxidation **(J)** measurement in the indicated HCT116 cells treated with 12 μM erastin for 26 h with or without 10 μM Lipro-1. **(K, L)** Cell death **(K)** and lipid peroxidation **(L)** measurement in the indicated MDA-MB-231 cells treated with 2 μM erastin for 12 h with or without 10 μM Lipro-1. **(M-P)** Cell death **(M, O)** and lipid peroxidation **(N, P)** measurement in the indicated HCT116 and MDA-MB-231 cells treated with cystine deprivation for 19h and 7h, respectively, with or without 10 μM Lipro-1. **A-P,** Data are the mean ± s.d.; n= 3 biologically independent experiments. Statistical analysis was performed using an unpaired two-tailed Student's t-test.

**Figure. S2.**


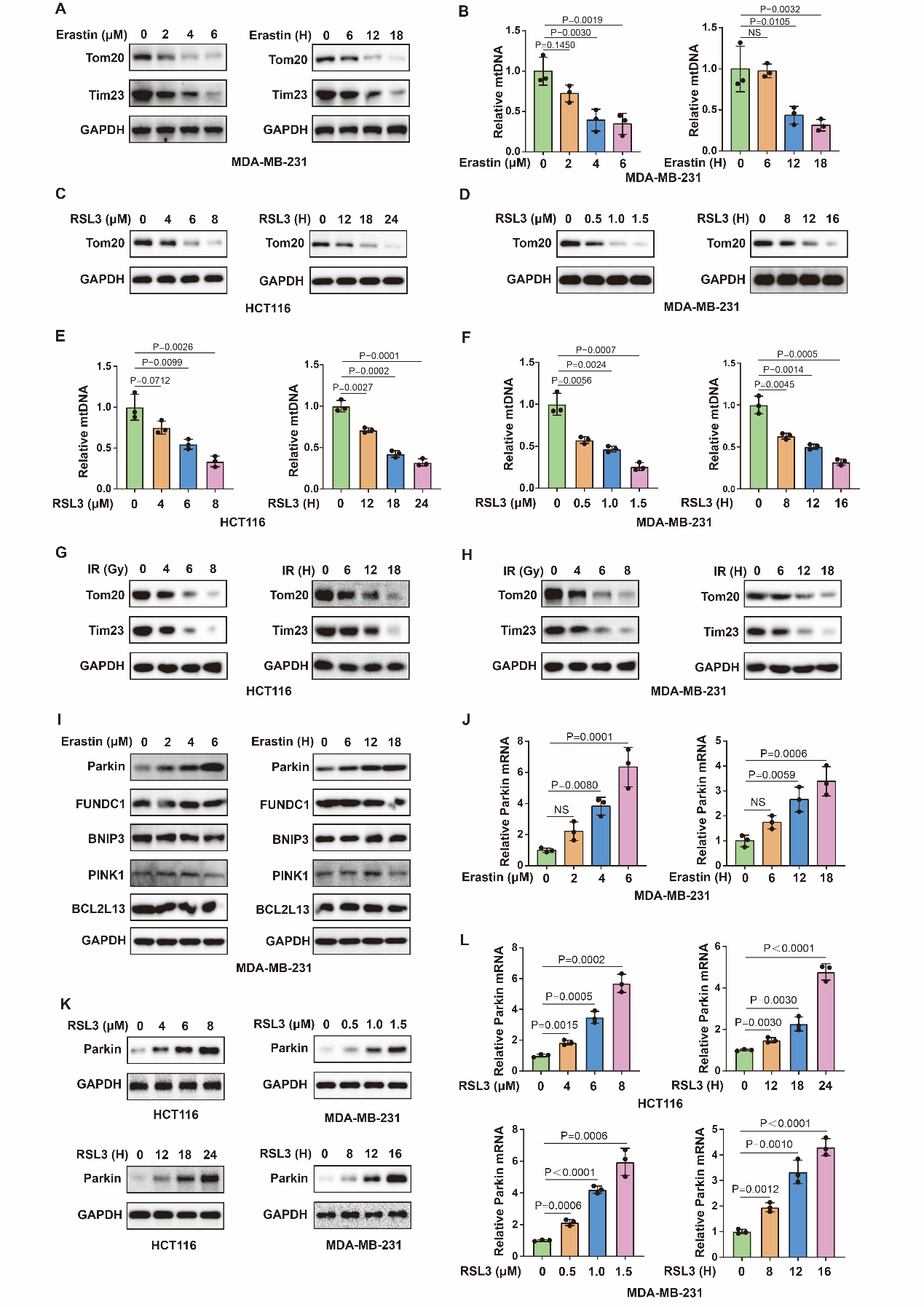


**Figure. S2. Increased Parkin expression promotes mitophagy during ferroptosis in cancer cells.** **(A)** Immunoblot showing the expression of Tom20 or Tim23 in MDA-MB-231 cells treated with erastin at the indicated concentrations and times. **Left,** time, 12h. **Right,** concentration, 3 μM. **(B)** The relative mitochondrial DNA (mtDNA) measurement in MDA-MB-231 cells treated with erastin at the indicated concentrations and times. **Left,** time, 12h. **Right,** concentration, 3 μM. **(C)** Immunoblot showing the expression of Tom20 in HCT116 cells treated with RSL3 at the indicated concentrations and times. **Left,** time, 18 h. **Right,** concentration, 6 μM. **(D)** Immunoblot showing the expression of Tom20 in MDA-MB-231 cells treated with RSL3 at the indicated concentrations and times. **Left,** time, 14 h. **Right,** concentration, 1 μM. **(E)** The relative mitochondrial DNA (mtDNA) measurement in HCT116 cells treated with RSL3 at the indicated concentrations and times. **Left,** time, 18 h. **Right,** concentration, 6 μM. **(F)** The relative mitochondrial DNA (mtDNA) measurement in MDA-MB-231 cells treated with RSL3 at the indicated concentrations and times. **Left,** time, 14 h. **Right,** concentration, 1 μM. **(G, H)** Immunoblot showing the expression of Tom20 or Tim23 in HCT116 **(G)** or MDA-MB-231 **(H)** cells treated with IR at the indicated intensities and times. **Left,** time, 18h. **Right,** intensity, 8 Gy. **(I)** Immunoblot showing the expression of mitophagy-related proteins in MDA-MB-231 cells treated with erastin at the indicated concentrations and times. **Left,** time, 12h. **Right,** concentration, 3 μM. **(J)** QPCR showing the expression of Parkin mRNA in MDA-MB-231 cells treated with erastin at the indicated concentrations and times. **Left,** time, 12h. **Right,** concentration, 3 μM. **(K, L)** Immunoblot **(K)** and QPCR **(L)** showing the expression of mitophagy-related proteins in HCT116 and MDA-MB-231 cells treated with RSL3 at the indicated concentrations and times. **A, C, D, G-I, K,** Data are representative of n= 3 biologically independent experiments. **B, E, F, J, L,** Data are the mean ± s.d.; n= 3 biologically independent experiments. Statistical analysis was performed using an unpaired two-tailed Student's t-test.

**Figure. S3.**

**
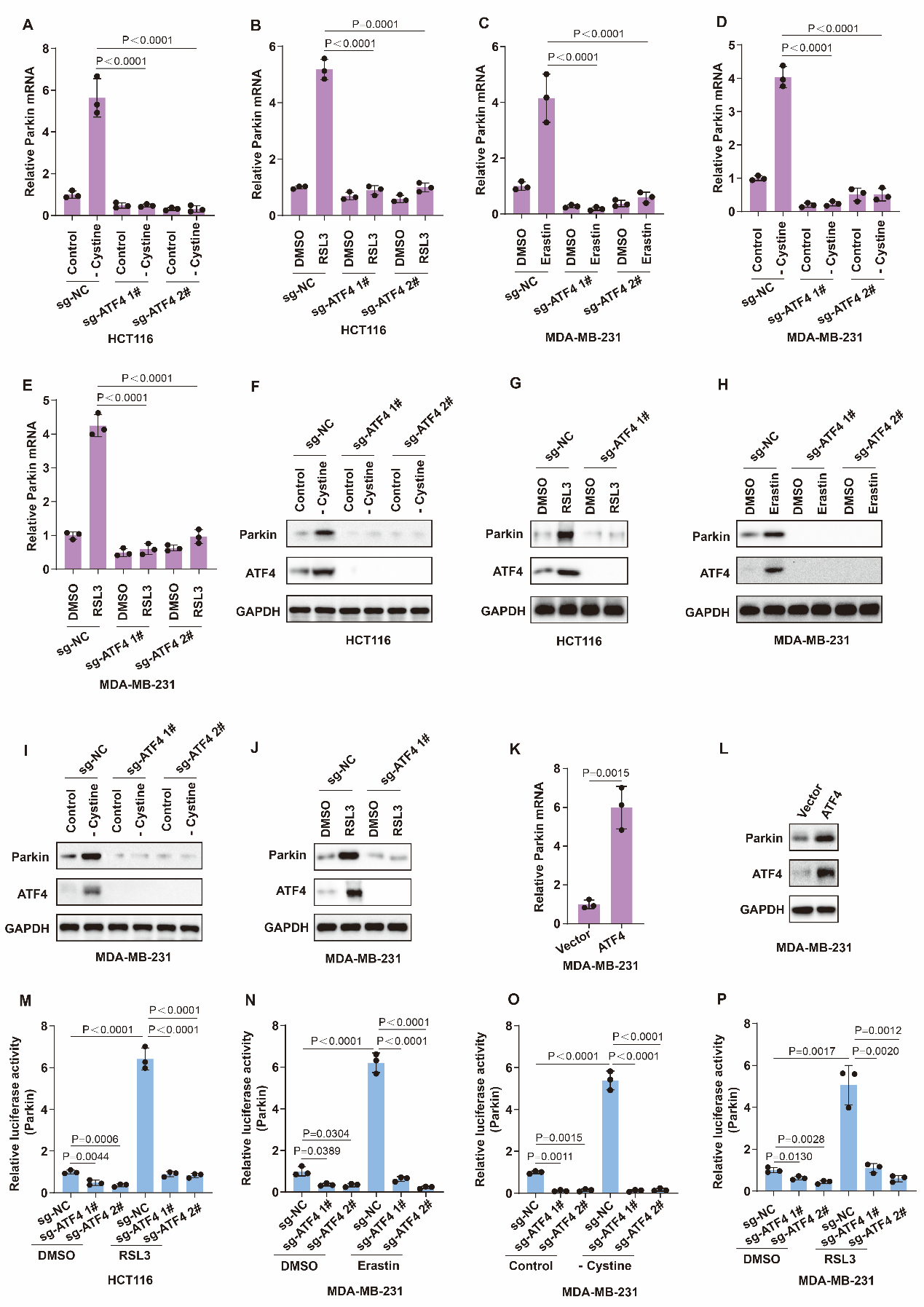
**

**Figure. S3. ATF4 transcriptionally up-regulates Parkin in cancer cells. (A)** Parkin mRNA measurement in control HCT116 cells (sg-NC) and ATF4 knockout HCT116 cells (sg-ATF4 1# or sg-ATF4 2#) treated with cystine deprivation for 16h. **(B)** Parkin mRNA measurement in control HCT116 cells (sg-NC) and ATF4 knockout HCT116 cells (sg-ATF4 1# or sg-ATF4 2#) treated with 6 μM RSL3 for 18 h. **(C)** Parkin mRNA measurement in control MDA-MB-231 cells (sg-NC) and ATF4 knockout MDA-MB-231 cells (sg-ATF4 1# or sg-ATF4 2#) treated with 2 μM erastin for 10 h. **(D)** Parkin mRNA measurement in control MDA-MB-231 cells (sg-NC) and ATF4 knockout MDA-MB-231 cells (sg-ATF4 1# or sg-ATF4 2#) treated with cystine deprivation for 6h. **(E)** Parkin mRNA measurement in control MDA-MB-231 cells (sg-NC) and ATF4 knockout MDA-MB-231 cells (sg-ATF4 1# or sg-ATF4 2#) treated with 1 μM RSL3 for 10 h. **(F)** Immunoblot showing the expression of Parkin and ATF4 in control HCT116 cells (sg-NC) and ATF4 knockout HCT116 cells (sg-ATF4 1# or sg-ATF4 2#) treated with cystine deprivation for 16h. **(G)** Immunoblot showing the expression of Parkin and ATF4 in control HCT116 cells (sg-NC) and ATF4 knockout HCT116 cells (sg-ATF4 1# or sg-ATF4 2#) treated with 6 μM RSL3 for 18 h. **(H)** Immunoblot showing the expression of Parkin and ATF4 in control MDA-MB-231 cells (sg-NC) and ATF4 knockout MDA-MB-231 cells (sg-ATF4 1# or sg-ATF4 2#) treated with 2 μM erastin for 10 h. **(I)** Immunoblot showing the expression of Parkin and ATF4 in control MDA-MB-231 cells (sg-NC) and ATF4 knockout MDA-MB-231 cells (sg-ATF4 1# or sg-ATF4 2#) treated with cystine deprivation for 6h. **(J)** Immunoblot showing the expression of Parkin and ATF4 in control MDA-MB-231 cells (sg-NC) and ATF4 knockout MDA-MB-231 cells (sg-ATF4 1# or sg-ATF4 2#) treated with 1 μM RSL3 for 10 h. **(K)** Parkin mRNA measurement in control MDA-MB-231 cells (Vector) and ATF4-overexpressing MDA-MB-231 cells (ATF4). **(L)** Immunoblot showing the expression of Parkin in control MDA-MB-231 cells (Vector) and ATF4-overexpressing MDA-MB-231 cells (ATF4). **(M)** Control HCT116 cells (sg-NC) and ATF4 knockout HCT116 cells (sg-ATF4 1# or sg-ATF4 2#) were transfected with Parkin-promoter firefly luciferase reporter construct and a constitutive-active Renilla luciferase reporter construct (pRL-CMV), and then treated with 6 μM RSL3 for 18 h. Relative luciferase activity was measured using the Dual-Luciferase Reporter Assay Kit (Promega E1980). **(N-P)** Control MDA-MB-231 cells (sg-NC) and ATF4 knockout MDA-MB-231 cells (sg-ATF4 1# or sg-ATF4 2#) were transfected with Parkin-promoter firefly luciferase reporter construct and a constitutive-active Renilla luciferase reporter construct (pRL-CMV), and then treated with 2 μM erastin for 10 h **(N)** or cystine deprivation for 6h **(O)** or 1 μM RSL3 for 10 h **(P)**. Relative luciferase activity was measured using the Dual-Luciferase Reporter Assay Kit (Promega E1980). **A-E, K, M-P,** Data are the mean ± s.d.; n= 3 biologically independent experiments. Statistical analysis was performed using an unpaired two-tailed Student's t-test. **F-J, L,** Data are representative of n= 3 biologically independent experiments.

**Figure. S4.**


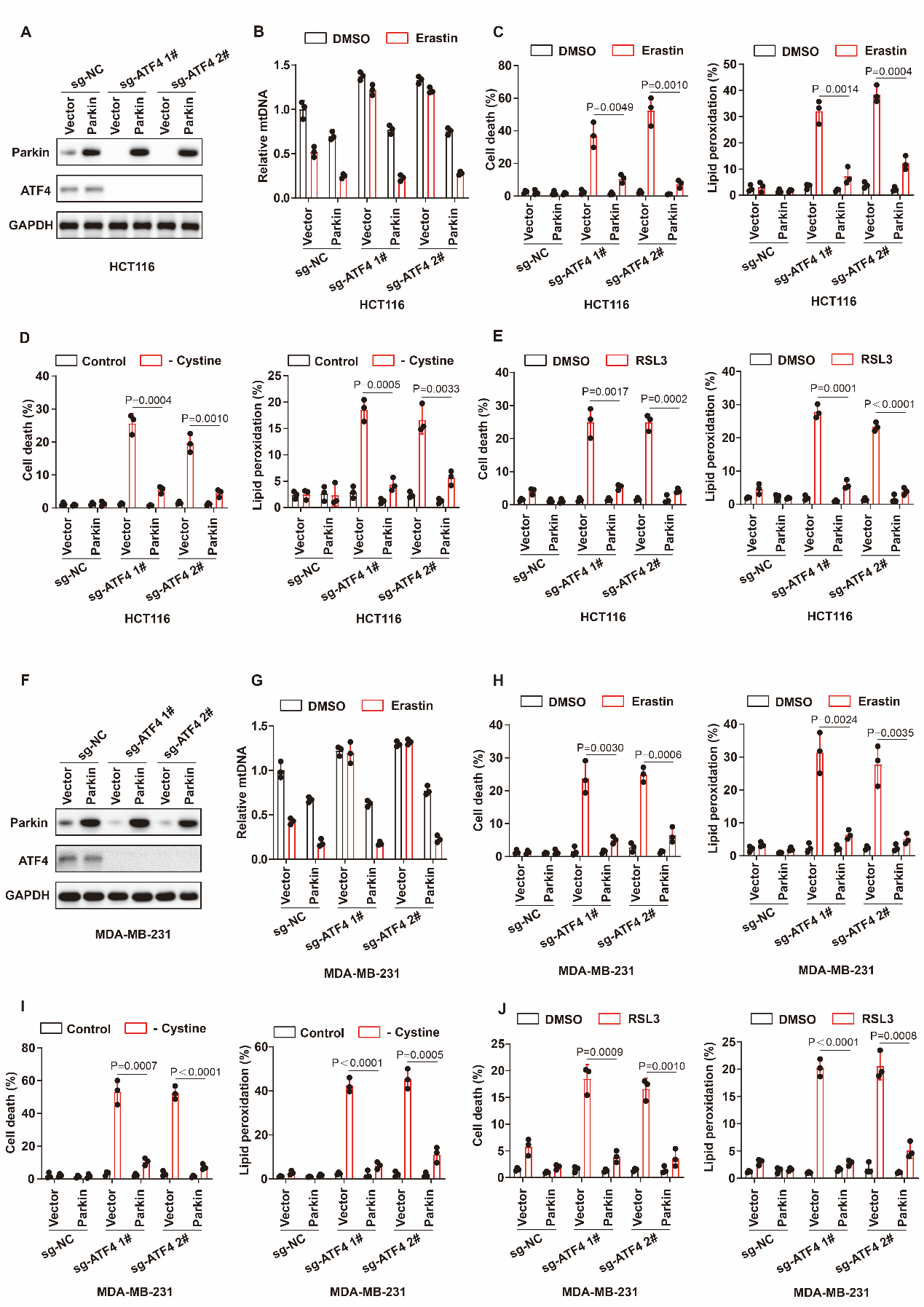


**Figure. S4. ATF4 transcriptionally up-regulates Parkin to regulate mitophagy and ferroptosis in cancer cells. (A)** HCT116 cells (sg-NC) and ATF4 knockout HCT116 cells (sg-ATF4 1# or sg-ATF4 2#) were stably transfected with empty vector (Vector) or Parkin plasmid (Parkin). Immunoblot showing the expression of Parkin and ATF4 in the indicated HCT116 cells. **(B)** The relative mitochondrial DNA (mtDNA) measurement in the indicated HCT116 cells treated with 12 μM erastin for 24h. **(C)** Cell death **(left)** and lipid peroxidation **(right)** measurement in the indicated HCT116 cells treated with 12 μM erastin for 25 h. **(D)** Cell death **(left)** and lipid peroxidation **(right)** measurement in the indicated HCT116 cells treated with cystine deprivation for 18h. **(E)** Cell death **(left)** and lipid peroxidation **(right)** measurement in the indicated HCT116 cells treated with 6 μM RSL3 for 18 h. **(F)** MDA-MB-231 cells (sg-NC) and ATF4 knockout MDA-MB-231 cells (sg-ATF4 1# or sg-ATF4 2#) were stably transfected with empty vector (Vector) or Parkin plasmid (Parkin). Immunoblot showing the expression of Parkin and ATF4 in the indicated MDA-MB-231 cells. **(G)** The relative mitochondrial DNA (mtDNA) measurement in the indicated MDA-MB-231 cells treated with 2 μM erastin for 10h. **(H)** Cell death **(left)** and lipid peroxidation **(right)** measurement in the indicated MDA-MB-231 cells treated with 2 μM erastin for 12 h. **(I)** Cell death **(left)** and lipid peroxidation **(right)** measurement in the indicated MDA-MB-231 cells treated with cystine deprivation for 7h. **(J)** Cell death **(left)** and lipid peroxidation **(right)** measurement in the indicated MDA-MB-231 cells treated with 1 μM RSL3 for 12 h. **A, F,** Data are representative of n= 3 biologically independent experiments. **B-E, G-J,** Data are the mean ± s.d.; n= 3 biologically independent experiments. Statistical analysis was performed using an unpaired two-tailed Student's t-test.

**Figure. S5.**


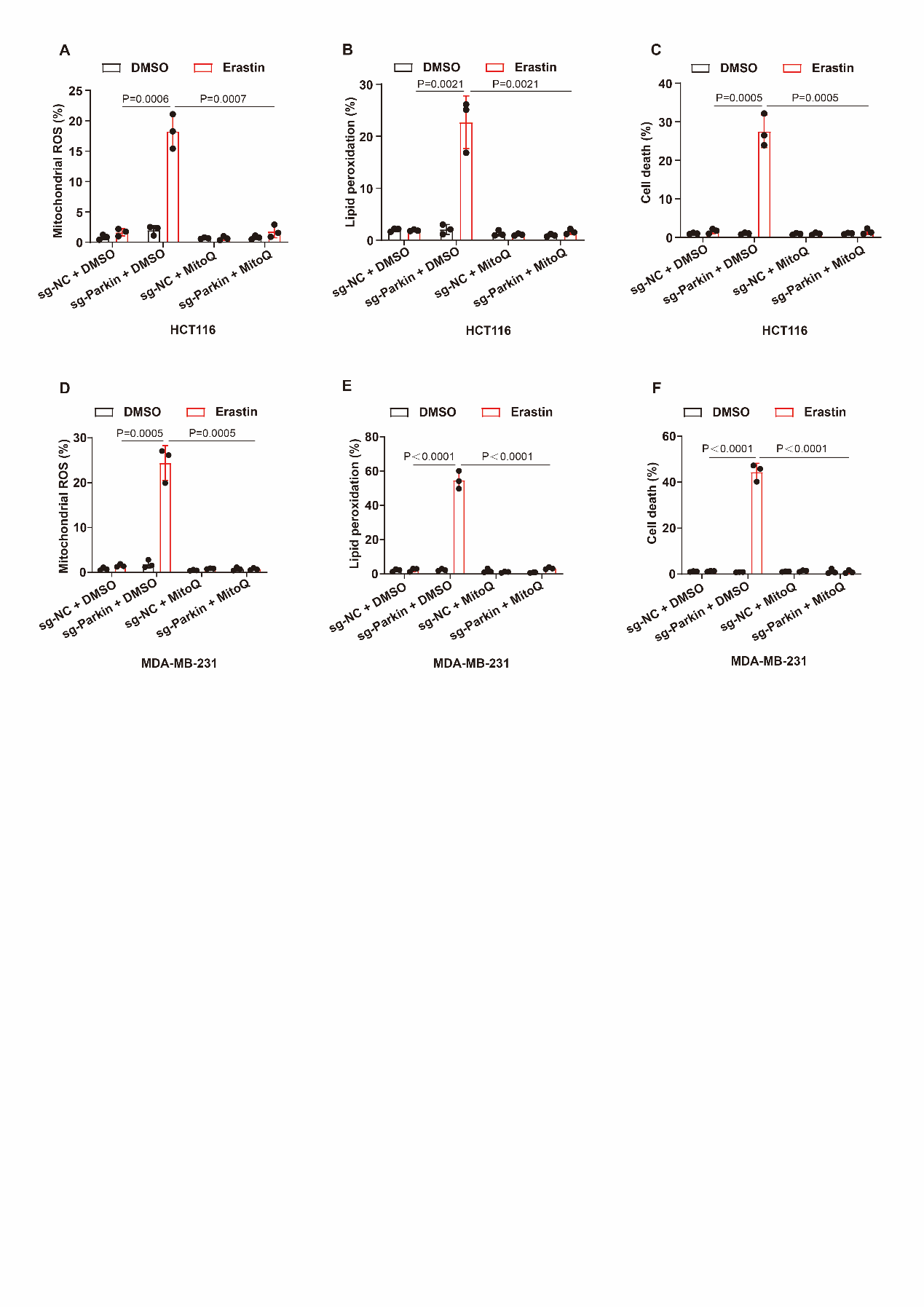


**Figure. S5. Mitophagy deficiency enhances cancer cell ferroptosis by increasing mitochondrial ROS.** **(A-C)** Mitochondrial ROS **(A)**, lipid peroxidation **(B)**, cell death **(C)** measurement in control HCT116 cells (sg-NC) and Parkin knockout HCT116 cells (sg-Parkin) treated with 12 μM erastin for 26 h with or without mitochondria-targeted ROS scavenger MitoQ. **(D-F)** Mitochondrial ROS **(D)**, lipid peroxidation **(E)**, cell death **(F)** measurement in control MDA-MB-231 cells (sg-NC) and Parkin knockout MDA-MB-231 cells (sg-Parkin) treated with 2 μM erastin for 12 h with or without mitochondria-targeted ROS scavenger MitoQ. **A-F,** Data are the mean ± s.d.; n= 3 biologically independent experiments. Statistical analysis was performed using an unpaired two-tailed Student's t-test.

**Figure. S6.**


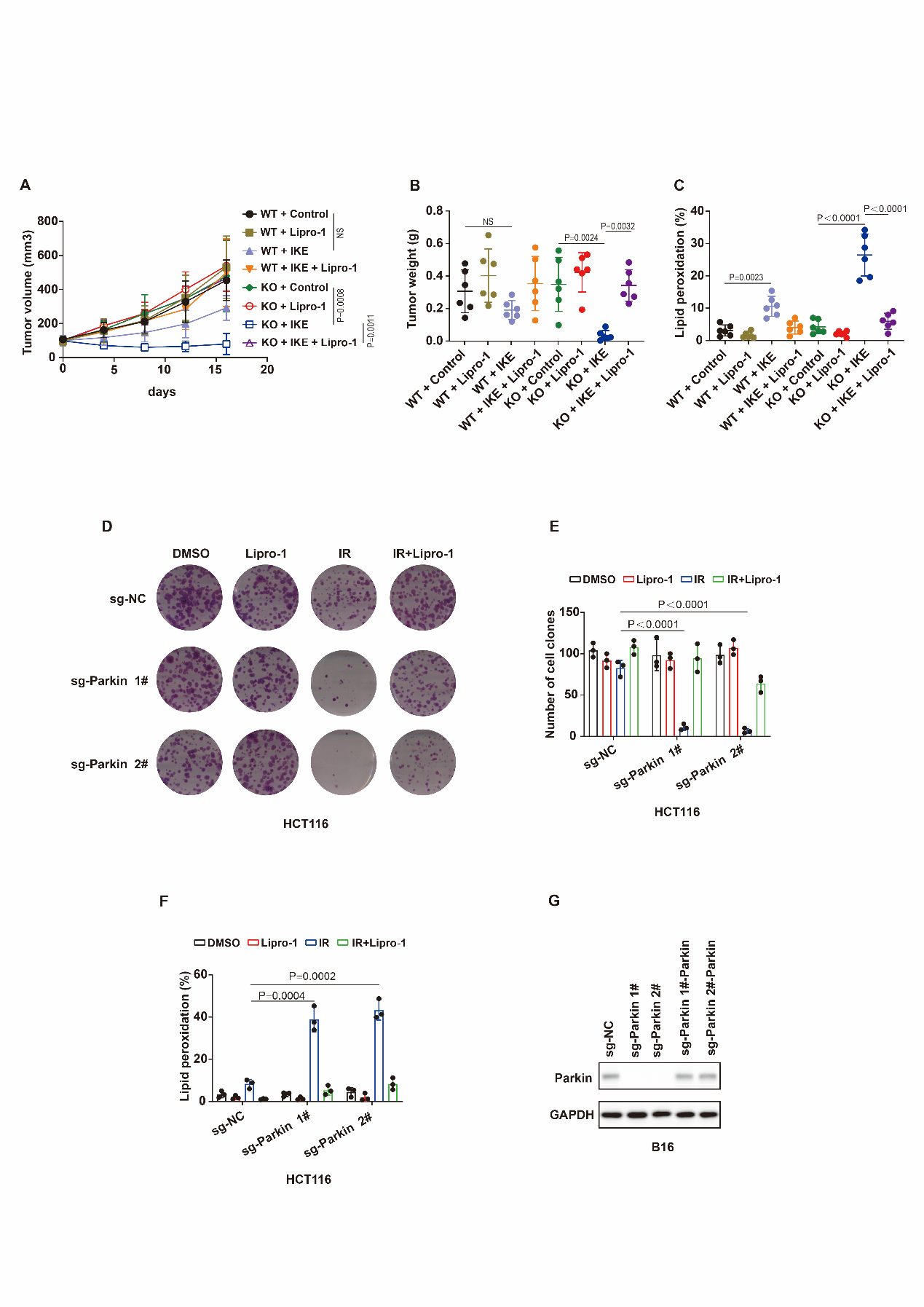


**Figure. S6. Mitophagy-deficient tumor growth is vulnerable to ferroptosis inducers.** **(A, B)** MDA-MB-231 cells were subcutaneously inoculated into BALB/c-nu nude mice. Mice were randomly assigned to different treatment groups 7 days after tumor inoculation. Imidazole ketone erastin (IKE) was injected intraperitoneally into mice at a dose of 30 mg/kg once daily for 15 days. Lipro-1 was administered three times before IKE followed by continued once daily administration at a dose of 15 mg/kg for 15 days. Tumor volumes **(A)** and tumor weights **(B)** of MDA-MB-231 xenograft tumors with the indicated treatments. **(C)** Relative lipid peroxidation in tumor cells isolated from the indicated tumors. **(D-F)** Cell clones **(D, E)** and lipid peroxidation **(F)** measurement in control HCT116 cells (sg-NC) and Parkin knockout HCT116 cells (sg-Parkin 1# or sg-Parkin 2#) treated with the indicated compounds or IR. Cell clones: IR, 4 Gy; lipro-1, 5 μM. Lipid peroxidation: IR, 8 Gy; lipro-1, 5 μM. **(G)** The expression of Parkin was restored in Parkin-knocked-out B16 cancer cells (sg-Parkin 1# or sg-Parkin 2#) to construct Parkin-restored B16 cancer cell lines (sg-Parkin 1#-Parkin 1 or sg-Parkin 2#-Parkin 1), and sg-NC was the control B16 cancer cell line. Immunoblot showing the expression of Parkin in the indicated B16 cells. **A,** Error bars are means ± SD, n = 6 independent repeats. P values were determined using 2-way ANOVA. **B, C,** Data are the mean ± s.d.; n= 6 biologically independent mice. Statistical analysis was performed using an unpaired two-tailed Student's t-test. **E, F,** Data are the mean ± s.d.; n= 3 biologically independent experiments. Statistical analysis was performed using an unpaired two-tailed Student's t-test. **G,** Data are representative of n= 3 biologically independent experiments.

**Figure. S7.**


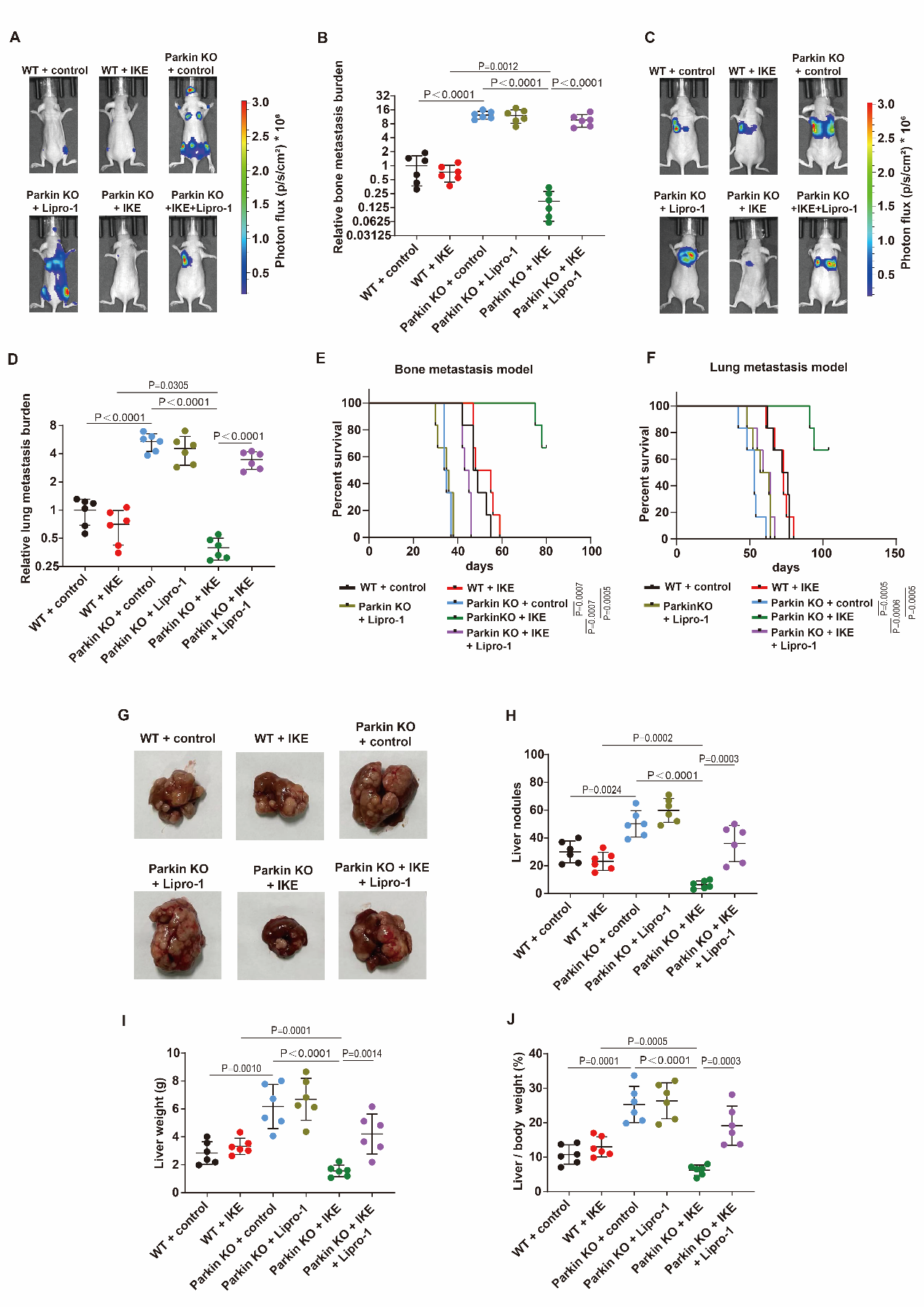


**Figure. S7. Mitophagy-deficient tumor metastasis is vulnerable to ferroptosis inducers. (A, B)** Mice were intracardiacally injected with wild-type MDA-MB-231 or mitophagy-deficient MDA-MB-231 cancer cells, then treated with control or IKE or Lipro-1 or IKE and Lipro-1. IKE was injected intraperitoneally into mice at a dose of 30 mg/kg once daily for 16 days starting from the day after cardiac injection of cancer cells. Lipro-1 was administered three times before IKE treatment followed by continued daily administration at a dose of 15 mg/kg for 16 days starting from the day after cardiac injection of cancer cells. The relative bone metastasis burden of each group of mice was measured. **(C, D)** Mice were tail-vein injected with wild-type MDA-MB-231 or mitophagy-deficient MDA-MB-231 cancer cells, then treated with control or IKE or Lipro-1 or IKE and Lipro-1. IKE was injected intraperitoneally into mice at a dose of 30 mg/kg every other day for 36 days starting from the day after tail vein injection of cancer cells. Lipro-1 was administered three times before IKE treatment followed by continued every other day administration at a dose of 15 mg/kg for 36 days starting from the day after tail vein injection of cancer cells. The relative lung metastasis burden of each group of mice was measured. **(E, F)** Survival time of the indicated bone metastasis mice **(E)** or lung metastasis mice **(F)** treated with control or IKE or Lipro-1 or IKE and Lipro-1. **(G-J)** Mice were injected intrasplenicly with wild-type MDA-MB-231 or mitophagy-deficient MDA-MB-231 cancer cells, then treated with control or IKE or Lipro-1 or IKE and Lipro-1. IKE was injected intraperitoneally into mice at a dose of 30 mg/kg every other day for 40 days starting from the day after the spleen injection of cancer cells. Lipro-1 was administered three times before IKE treatment followed by continued every other day administration at a dose of 15 mg/kg for 40 days. The liver nodules **(G, H)**, liver weight **(I)**, liver/body weight ratio **(J)** in the indicated mice was measured. **A-J,** Data are the mean ± s.d.; n= 6 biologically independent mice. Statistical analysis was performed using an unpaired two-tailed Student's t-test.
